# Two in one: a randomized controlled trial on an internet-based intervention (Lenio) for management of both chronic pain and depressive symptoms

**DOI:** 10.3389/fpsyt.2025.1528128

**Published:** 2025-03-18

**Authors:** Swantje Borsutzky, Anna-Sophie Wilke, Josefine Gehlenborg, Steffen Moritz

**Affiliations:** Department of Psychiatry and Psychotherapy, University Medical Center Hamburg-Eppendorf, Hamburg, Germany

**Keywords:** chronic pain, depression, e-mental health, self-guided internet-delivered intervention, self-help application, unguided iCBT, iCBT

## Abstract

The high prevalence of chronic pain and comorbid depression, along with their negative impacts on individuals and society, highlights the need for accessible and effective interventions. This study examined the feasibility, efficacy, and acceptability of Lenio, an Internet-based self-help intervention, combined with the COGITO smartphone application for managing these conditions. The randomized controlled trial involved an intervention group (IG) using Lenio and COGITO, an active control group (ACG) using a transdiagnostic app, and a wait-list control group (WCG) receiving usual treatment. Lenio incorporates cognitive behavioral therapy and third-wave techniques, supported by COGITO’s gamified daily exercises to enhance adherence. Across 263 participants assessed at three intervals over 16 weeks, the IG showed significant improvement in somatic-affective depressive symptoms compared to the WCG post-intervention and both control groups at follow-up. However, the ACG outperformed the IG in managing pain at post-intervention though these effects did not persist until follow-up. The interventions were well-received and feasible, with older participants benefiting more from the Lenio/COGITO combination. Lenio and COGITO could bridge gaps to conventional therapy, potentially easing pain by enhancing psychological well-being and coping strategies. Future studies should investigate tailored interventions for chronic pain and their long-term effectiveness.

**Protocol:**

Full trial protocol can be accessed via DOI: 10.1186/s13063-023-07440-8.

**Clinical trial registration:**

https://drks.de/register/de/trial/DRKS00026722/preview, identifier DRKS-IDDRKS00026722.

## Introduction

### Pain and psyche

The fact that the body and mind mutually influence each other is not a new insight but has been suspected since ancient times. In line with this, studies have found that chronic pain and comorbid depression influence each other (1). First, on a neuronal level, brain areas and neuronal pathways related to pain are often implicated in the experience of negative emotions or depression (2). Added to this, increased cognitive engagement (e.g., brooding, focus on pain), self-esteem problems, and catastrophizing are frequently seen in individuals affected by chronic pain (3). These thinking patterns become entrenched over time, thus compromising mental health and intensifying the pain experience (3). Although the interactions between chronic pain and psychological well-being have been recognized for a long time and psychotherapy is increasingly acknowledged as part of the gold-standard multimodal treatment (4), psychoeducational and psychotherapeutic interventions are still rarely utilized in conventional pain management due to significant treatment gaps (5). Consequently, many patients with chronic pain are left to manage their symptoms alone (6), resulting in up to 85% of these individuals experiencing depressive symptoms in addition to their pain (7). Studies show that chronic pain is one of the most common reasons people seek medical support, with a global prevalence of up to 24% (8). Pharmacological treatments are often used as a standalone approach for managing both chronic pain and depression. However, while medication may alleviate pain to some extent, it does not address the underlying psychological and behavioral factors contributing to the chronic nature of these conditions (9, 10).

### Psychotherapeutic treatment of chronic pain

Research has shown considerable improvement in chronic pain following psychotherapeutic interventions. A meta-analysis by Veehof et al. (11) revealed that acceptance- and mindfulness-based interventions led to notable decreases in chronic pain intensity and interference compared to control groups receiving no treatment, medical treatment as usual, or educational and support interventions. Cognitive behavioral therapy (CBT) and its third wave techniques are considered the gold standard in psychotherapeutic treatment of individuals with chronic pain (12, 13). Despite this strong evidence, psychotherapeutic intervention in pain is still the exception rather than the rule (6, 14). Many people with chronic pain are themselves unaware of the connection between pain and psychological factors (15). Furthermore, affected people often struggle with low self-efficacy and depressive symptoms, which can make it difficult for them to take initiative and seek therapy (16). Immobility can also be a factor for pain patients, making it more difficult for them to access healthcare facilities, hospitals, and therapists (17, 18).

These arguments underscore the need for low-threshold, accessible, and effective treatment options that meet the needs of people with chronic pain and comorbid depression.

### Internet-based self-help programs

Due to the inherent flexibility and anonymity of Internet-based interventions, compliance in studies is usually high (19, 20). Internet-based programs can be offered unguided (self-help) or guided (e.g., by a therapist). A meta-analysis by Carlbring et al. (21) showed that guided Internet-based interventions for psychiatric and somatic disorders (e.g., OCD, anxiety, fatigue, chronic pain) have a similar effect size to conventional face-to-face therapy. If the main drawback of unaccompanied interventions—adherence to the program (22)—is addressed, they can provide an alternative to conventional therapy in the treatment of chronic pain (21, 23, 24) and are somewhat superior to guided interventions in terms of resource efficiency and anonymity (20).

### Self-help programs for chronic pain

Buhrman and colleagues conducted a systematic review of Internet-based interventions aimed at addressing chronic pain and concluded that these programs have a modest impact on the reduction of pain intensity (Hedges’s *g* = − 0.33 (25)). To the best of our knowledge, however, there is currently no freely accessible, Internet-based self-help program specifically designed for the treatment of chronic pain and comorbid depressive symptoms in Germany. This led us to research and develop such an intervention.

In a pilot study, Miegel and colleagues (26) conducted an evaluation of a guided Internet-based intervention against depression called NOVEGO in people with chronic pain. Significant reductions in depressive symptoms with small to moderate effect sizes emerged in the NOVEGO group compared to the wait-list control group (η_p_^2^ = 0.043). However, improvements in pain intensity were only evident among a subgroup, including those experiencing pain in their upper back or hands. Thus, there is a need for an Internet-based program that specifically caters to the unique requirements of patients dealing with chronic pain and comorbid depressive symptoms. These requirements include accessibility for patients with limited mobility, flexibility to adapt treatment to the patients’ schedules, and anonymity to reduce stigma or hesitation in seeking help. Furthermore, such an intervention should offer tailored psychoeducational content that addresses the interaction between chronic pain and psychological factors and offers evidence-based techniques, particularly cognitive behavioral and mindfulness-based approaches, to improve coping strategies and self-efficacy. Our intervention, Lenio and the accompanying smartphone application COGITO, aim to address these needs by providing a low-threshold, self-paced Internet intervention designed specifically for this target group.

The objective of the present study was to evaluate the feasibility, efficacy, and acceptance of the Internet-based self-help intervention Lenio combined with COGITO in addressing the particular requirements of people with chronic pain and comorbid depressive symptoms, which include accessibility, flexibility, and tailored psychoeducational content.

## Methods

### Study design

In this three-armed randomized controlled trial (RCT) with three measurement time points, an online assessment was conducted at baseline (t0), followed by an 8-week online post assessment (t1) and a 16-online-based follow-up assessment (t2). The design allowed for the exploration of underlying mechanisms by comparing three different treatment approaches—an intervention group (IG) receiving Lenio/COGITO, a waitlist control group (WCG) receiving treatment as usual, and an active control group (ACG) receiving an alternative app—and their effects over time. Participants provided informed consent through the web platform during the baseline assessment. Throughout the study, no personally identifiable information was collected, and participants were asked to create a pseudonymous email address (participants were provided instructions for this) and a personal code-word. All collected data were pseudonymized and securely stored electronically on a password-protected computer. Participants had the option to request the deletion of their data by providing either their code-word or their pseudonymized email address. At the end of the post-assessment period, participants in the IG, WCG, and ACG were provided with a gift card for various online shops as an incentive and then also received access to Lenio and the COGITO app. The study was conducted in accordance with the principles outlined in the Declaration of Helsinki. The study was registered at the German Clinical Trials Register (DRKS00026722). The local psychological ethics committee at the Center for Psychosocial Medicine, University Medical Center Hamburg-Eppendorf (Germany), approved the study project (approval number: LPEK-0078a).

### Participant recruitment

Participants were recruited from inpatient and outpatient clinics in Germany. Additionally, study details and links were shared through relevant social media groups, such as groups for chronic pain. A targeted advertising campaign was implemented using Facebook/Instagram^®^, and Google ads. Further recruitment sources included self-help groups, Internet forums, websites, newsletters of health insurance companies/associations, and various social media platforms (Instagram, Reddit, Twitter, YouTube, LinkedIn). This approach aimed to reach not only patients already in treatment but also individuals from the wider community. Participants received comprehensive information about the study’s objectives, procedures, and data protection measures at the beginning of the baseline assessment. Electronic informed consent was obtained from each participant.

### Participant selection criteria

To be included in the RCT, participants had to meet the following criteria: (a) presence of depressive symptoms (Beck Depression Inventory-II (BDI-II) score ≥ 14; or Patient Health Questionnaire-9 (PHQ-9) score ≥ 10), (b) presence of chronic pain symptoms (mean score pain intensity on the German Pain Questionnaire (DSF) ≥ 4), (c) age between 18 and 75 years, (d) provision of informed consent, (e) sufficient command of the German language, (f) willingness to participate in three anonymous online surveys, (g) willingness to use the Internet-based treatment program for a minimum of 8 weeks, and (h) access to a computer/laptop and a smartphone.

The program was designed to cater to a diverse group of patients with chronic pain and co-occurring depressive symptoms. The effects of previous diagnoses and concurrent treatment programs were analyzed through moderation analyses.

**Figure 1:**
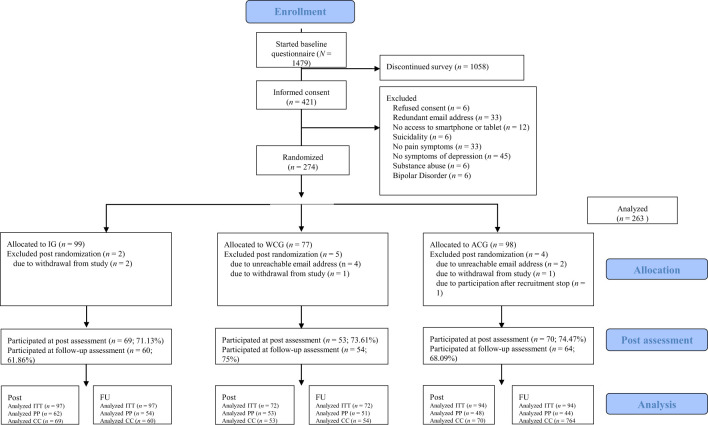
CONSORT flow chart.

Exclusion criteria included a lifetime diagnosis of schizophrenia spectrum disorder, bipolar disorder, substance abuse disorder, or acute suicidality (assessed using an item on suicidality in the Web Screening Questionnaire; WSQ). Inclusion and exclusion criteria were assessed during the baseline assessment. Excluded participants were provided with an explanation for their exclusion and received information about alternative resources for seeking help, including telephone numbers for acute crisis situations. Data collection for the study took place in Germany from November 2021 (first baseline assessment) to August 2022 (last follow-up assessment). A total of 147 participants were excluded as they did not meet the inclusion criteria. The final sample included 263 individuals (see **Figure 1**).

### Procedure

Data were collected at each measurement point (baseline, post, and follow-up) using the survey software Qualtrics^®^. During the baseline assessment, participants’ sociodemographic information and psychopathological data were assessed and the participants filled out questionnaires on their cognitions and beliefs about their symptoms (see *Measures* section for details). After baseline, participants were randomized into one of three groups: IG, ACG, or WCG. During the 8-week intervention period, the IG used the Internet-based self-help intervention with an accompanying smartphone app and the ACG used a self-help smartphone app without pain-specific content (for more detailed descriptions, see interventions). After the 8-week intervention period, all participants received an email invitation to participate in the post assessment. They were required to enter their pseudonymous email address and personal code again to ensure accurate matching of pre-intervention and post-intervention data. This process was repeated after 16 weeks for the follow-up assessment.

During the post assessment and follow-up assessment, participants completed the same psychopathological questionnaires as those used in the baseline assessment. Additionally, participants were asked how frequently they had used the intervention (“How often have you used Lenio during the past 8 weeks?”), any side effects they experienced, and their satisfaction with the self-help intervention (see *Measures* section for details). The study was conducted at the Hamburg-Eppendorf University Medical Center in Germany.

### Randomization

Randomization was conducted using the Qualtrics survey software following the baseline assessment. The “equal distribution” option was selected to ensure a balanced distribution among the three groups. The allocation rule was set to 1:1:1; that is, participants were assigned to each group in an equal ratio.

### Sample size

The sample size calculation for an analysis of covariance (ANCOVA) with the three groups was conducted using G*Power. The results indicated a required sample size of 246 participants, assuming an effect size of η_p_^2^ = 0.06 (moderate effect), a significance level of α = 0.05 (two-sided), and a power of 0.95. Anticipating a dropout rate of 25%, we aimed to recruit 300 participants, with 100 participants assigned to each condition. This calculation was based on the aforementioned findings from a study (26) that examined the impact on patients dealing with chronic pain of an unguided Internet-based intervention for depression that did not include pain-related content and reported small to moderate effects.

### Measures

#### Primary outcomes

The primary outcome measure was pain interference, which encompasses the average level of impairment experienced in daily life, leisure time, and work as evaluated by the German Pain Questionnaire (DSF). The DSF is a reliable and valid self-report questionnaire and is widely used for assessing pain symptoms (27, 28). The practicality and content validity of the questionnaire have been rigorously evaluated (29). The German Pain Society advises using the DSF initially and during pain therapy as a standardized tool for quality assurance (29). It evaluates pain across multiple dimensions, such as pain location, subjective description, onset and course of pain, pain attacks, pain intensity, duration of illness, and pain-related impairment in everyday life. The DSF also captures information on previous treatments, rehabilitation clinic stays, healthcare visits, surgeries, and comorbid illnesses. Certain items assessing well-being, anxiety, and depression were excluded from the survey as these dimensions were tapped by other questionnaires (e.g., BDI-II, PHQ-9). Pain interference was calculated based on the von Korff disability score, and pain intensity (as a secondary measure) was calculated based on the von Korff index (30).

#### Secondary outcomes

Secondary outcome measures included depressive symptoms and pain symptom severity. Additionally, participants filled out a subjective evaluation of the intervention.

##### BDI-II

The BDI-II is a self-report questionnaire consisting of 21 items assessing depressive symptoms experienced over the previous two weeks (31). Scores range from 0 to 63, with higher scores indicating greater levels of depression. The BDI-II demonstrates good internal consistency, with a Cronbach’s α of 0.89 (32) and has been previously used in pain patient samples (33, 34). For this study, we divided the BDI-II into two subscales based on the findings of Huang & Chen (35): somatic-affective and cognitive. This division allows for a more nuanced understanding of depression by separately examining its emotional-physical and thought-related components.

##### PHQ-9

The PHQ-9 is a self-report questionnaire that measures the severity of depressive symptoms over the preceding week (36, 37). It consists of nine items and demonstrates high internal consistency (Cronbach’s α = 0.86–0.89). Scores on the PHQ-9 range from 0 to 27, with higher scores indicating more severe depression. The sum score of the PHQ-9 was calculated for each assessment point.

##### Web screening questionnaire (WSQ)

The WSQ is a brief online self-report tool designed to screen for common mental disorders, including affective disorders, anxiety disorders, posttraumatic stress disorder, and suicide risk (38). Sensitivity and specificity of the WSQ vary between 0.72 and 1.00 and 0.44 and 0.77, respectively (38). The WSQ was administered at baseline, and both the sum score and cumulative values for all subscales were calculated for analysis purposes (e.g., moderation analysis).

##### Injustice experience questionnaire (IEQ)

The IEQ is a reliable and valid tool for assessing perceived injustice in individuals with chronic pain (3). It consists of 12 items that measure unfairness related to symptoms on a 5-point scale. The elements examined include severity of loss, blame, sense of unfairness, and irreparability of loss. A score of 30 indicates a clinically relevant level of perceived injustice. The subscales “blame/injustice” and “severity/irreparability” and the total score were calculated at all three measuring points.

##### Pain catastrophizing scale (PCS)

The PCS is a reliable and valid measure of catastrophizing in patients with chronic pain. It includes 13 items assessing thoughts and feelings during pain on a 5-point scale. Quartana and colleagues found good internal consistency, with a Cronbach’s α of 0.87 (39). The PCS total score and subscores for “Rumination,” “Helplessness,” and “Magnification” were calculated at all three measuring points.

##### Fear-avoidance beliefs questionnaire (FABQ)

The FABQ was originally developed after the emergence of the biopsychosocial model for understanding low back pain by Waddell and colleagues (40). It assesses fear-avoidance beliefs about physical activity and its relation to chronic pain. It consists of 16 items rated on a 7-point Likert scale. The FABQ demonstrates excellent test-retest reliability (ICC = 0.97 (41);. For this study, the FABQ was adapted to assess general chronic pain. The sum scores for “bodily activity” and “workload” were calculated at all three measuring points.

##### Pain self-efficacy questionnaire (FSS)

The Pain Self-Efficacy Questionnaire is a valid instrument for assessing self-efficacy in pain research. It measures an individual’s belief in their ability to engage in activities despite pain. It has high internal consistency, with a Cronbach’s α of 0.93 (42). The sum scores for the subscales “active coping” and “catastrophizing” were assessed at all three measuring points.

##### World health organization quality of life abbreviated version (WHOQOL-BREF)

The WHOQOL is a cross-cultural questionnaire that assesses generic quality of life. It considers an individual’s perception of their life and the context of culture and value systems (e.g., personal goals). We used the global QoL item, ranging from “very poor” to “very good.” The global item was assessed at all measuring points.

### Subjective evaluation

The participants’ subjective evaluation of Lenio and COGITO was measured using the Client Satisfaction Questionnaire (CSQ-8). Previous research indicates that the CSQ-8 has high psychometric properties, with internal consistency ranging from Cronbach’s α = 0.87 to 0.93 (43). Participants rated their satisfaction on a 4-point Likert scale (“excellent”, “good”, “less good”, “bad”), where a higher score indicated greater satisfaction. The questionnaire was modified to replace “psychotherapy” with “self-help intervention Lenio”.

Data on subjective appraisal of the intervention was collected through 12 questions assessing the quality, utility, and applicability of Lenio and COGITO using both open and closed response formats. The responses to the seven closed questions was assessed on a 4-point Likert scale (“not applicable” to “completely true”). The open-ended questions gathered positive and negative feedback on Lenio and requested suggestions for improvement. Additionally, participants were asked to indicate the frequency of Lenio usage during the intervention period. Subjective data were collected at post-intervention and follow-up assessments.

### Interventions

During the intervention period, the IG had access to the Internet-based self-help intervention Lenio and accompanying self-help app COGITO. The ACG had access to the self-help smartphone app MCT & More, a forerunner version of COGITO intended for individuals with depressive symptoms with no specific pain-related content. The WCG received treatment as usual.

### Lenio

The self-help Internet-based intervention Lenio consists of a welcome module, an introduction module, and nine modules targeting chronic pain and comorbid depressive symptoms, including dysfunctional coping. Some modules cover broader topics such as social competence and self-worth, while others focus specifically on chronic pain using techniques such as acceptance and commitment therapy (ACT), addressing specific needs, and relapse prevention.

Modules are further divided into subunits, allowing participants to prioritize topics or skip familiar ones. Users have flexibility in choosing the module order, and they can pause at any time and easily resume where they left off. Completing at least two modules per week was recommended, with each module taking 30 to 60 minutes on average.

Upon logging in, participants are greeted with an introductory video and then proceed to the welcome module, which utilizes motivational interviewing techniques and interactive dialogues with avatars. Throughout the program, psychoeducational content, interactive exercises, worksheets, graphics, videos, and audios are provided. A moderator is available for technical assistance, responding within three workdays, but does not offer therapeutic guidance. Lenio offers an accessible and anonymous platform for individuals seeking assistance in managing their condition. To enhance its effectiveness, we recommended that Lenio be used in conjunction with the smartphone app COGITO, combining the advantages of an Internet-based innovation with the benefits of a mobile (smartphone) app (e.g., regular dispatch of notifications to increase usage).

### COGITO

COGITO is a smartphone app that can be downloaded from the Google Play Store (for Android® users) and the App Store (for iOS® users). Download links are also available through the Lenio desktop app. In the introduction module, participants are instructed on how to use the app. COGITO enhances usability by sending daily push notifications with short exercises (maximum reading time of 30 seconds) and incorporating gamification elements, such as collecting medals for completed exercises or action scores for total weekly usage. Participants have the flexibility to choose the time and frequency of push notifications. COGITO offers exercise packages entitled Mood and Self-Esteem, Psychosis, Gambling Problems, OCD, Sleep and Chronic Pain. Participants were explicitly encouraged to activate the Chronic Pain and Mood and Self-Esteem packages, while other packages were initially deactivated but could be individually activated by users. Similar to Lenio, COGITO exercises are based on CBT and third-wave techniques. Previous randomized controlled trials (RCTs) of an earlier version of the COGITO app without a pain program package demonstrated significant improvements in self-reported depressive symptoms and increased self-esteem among regular users compared to individuals on a wait-list (44, 45).

### MCT & More

The ACG had access to the MCT & More smartphone app, which is a pilot version of the COGITO app and shares a similar design and concept. MCT & More consists of three packages focusing on mood, metacognitive training (MCT), and gambling. However, it does not include pain-specific exercises. Participants in the ACG were allowed to choose the packages from which they wanted to receive exercises.

### Adherence measures

To promote adherence to the recommended pace of completing two modules of Lenio per week, we implemented several supportive measures. Participants received regular reminder emails that highlighted specific modules that might be of interest to them, encouraging continued engagement with the program. Furthermore, the accompanying smartphone application COGITO sent daily push notifications prompting participants to complete an exercise. These measures aimed to foster regular interaction with the intervention while allowing participants the flexibility to proceed at their own pace.

Adherence to the recommended schedule was voluntary throughout the study, however, and depended on individual preferences. Retrospective analysis of module completion data provides insight into the extent of participant engagement, as reported in the results section.

### Statistical analysis

IBM Statistics 29® was utilized for conducting the statistical analysis. ANCOVAs with difference scores (pre-post and pre-follow-up differences) as the dependent variable were calculated. Groups represented the between-group factor. The baseline score of the dependent variable served as covariate. Paired samples *t*-tests were employed to analyze within-group differences. Independent samples *t*-tests were employed to compare baseline characteristics between groups.

To assess the efficacy of Lenio and COGITO, three types of analyses were performed: intention-to-treat (ITT), per-protocol (PP), and complete-case (CC). In ITT analyses, all participants with available baseline data were included in the evaluation. Missing post values were estimated using expectation maximization. PP analyses included only those participants who used the intervention as intended (at least once a week) and completed the post assessment. CC analyses included all participants who completed the post or follow-up assessment, regardless of whether the intervention was used. The CONSORT guidelines recommend performing both ITT and PP analyses in randomized controlled trials (46, 47). ITT analyses adhere to the conservative approach of good clinical practice and are considered the gold standard for evaluating treatment effects. PP analyses provide an estimation of actual efficacy under ideal conditions.

Additionally, an exploratory moderation analysis was conducted for the PP sample using the SPSS macro PROCESS by Hayes (48). This analysis aimed to identify potential moderators (including sociodemographic data, psychometric scales, and medication that influenced differential symptom improvement as measured by the DSF (von Korff) and the BDI-II).

## Results

The final sample included 263 participants who were evenly randomized (1:1:1) to the IG (*n* = 97), the WCG (*n* = 72), or the ACG (*n* = 94; see **Figure 1** for the study flowchart).

### Sample description

Demographic and psychopathological characteristics at baseline are presented in **Table 1**. The sample averaged 43.70 years (*SD* = 13.66) of age and was predominantly female (*n* = 209, 79.5%), with no significant differences between the three groups on any variables, confirming successful randomization.

**Table 1:** Sociodemographic characteristics and psychopathology at baseline.

Variable	IG Lenio*/*COGITO (*n* = 97)	ACG (*n* = 94)	WCG (*n* = 72)	Statistical Analysis
Demographics				
Gender (male/female/diverse)	15 (15.5%) / 80 (82.5%) / 2 (2.1%)	17 (18.1%) / 76 (80.9%) / 1 (1.1%)	18 (25.0%) / 53 (73.6%) / 1 (1.4%)	χ^2^ = 2.810 (4), *p* = .590, η_p_^2^ = .097
Age in years	42.08 (13.42)	44.20 (13.85)	45.25 (13.71)	*F* (2,260) = 1.208; *p* = .300, η_p_^2^ = .009
Number of years in school	12.41 (2.07)	11.97 (1.72)	12.35 (2.49)	*F* (2,260) = 0.012; *p* = .988, η_p_^2^ = .000
Psychopathology				
BDI-II	26.20 (19.76)	25.41 (9.83)	25.71 (9.34)	*F* (2,260) = 0.147; *p* = .864, η_p_^2^ = .001
PHQ-9	13.84 (5.80)	13.47 (5.10)	12.82 (4.52)	*F* (2,260) = 0.785; *p* = .457, η_p_^2^ = .006
Von Korff severity score	3.52 (2.13)	3.11 (1.06)	2.97 (1.05)	*F* (2,260) = 1.480; *p* = .230, η_p_^2^ = .011
Current treatment				
Currently on pain medication	66 (68.0%)	58 (61.7%)	42 (58.3%)	χ^2^ = 2.197 (2), *p* = .333, η_p_^2^ = .091
Currently in psychotherapeutic treatment	56 (57.7%)	51 (54.3%)	38 (52.8%)	χ^2^ = 0.456 (2), *p* = .796, η_p_^2^ = .042

IG, Intervention group receiving Lenio and COGITO; WCG, Waitlist control group receiving TAU; ACG, Active control group receiving MCT & More app: BDI – II, Beck Depression Inventory-II; PHQ-9, Patient Health Questionnaire-9.

### Study completion and intervention usage

Of the 263 participants, 193 participated in the post assessment (73.35%) and 178 participated in the follow-up assessment (67.52%), with no significant differences across groups (Lenio/COGITO 71.13% at post and 61.86% at follow-up, WCG 73.61% at post and 75% at follow-up, ACG 74.47% at post and 68.09% at follow-up). On average, participants reported having completed 1.95 modules (out of 10; *SD* = 2.85). Mean usage duration of Lenio and the COGITO app was *M* = 3.30 (on a scale from 1 = “used only once” to 7 = “daily”; 3 = less than one time per week; *SD* = 2.08).

### Between-group differences

#### ITT and PP analyses

All analyses are displayed in the Appendix. ITT analyses showed a significant between-group difference in pain interference symptoms, as indexed by the von Korff disability score (primary outcome; *F (2*,259) = 3.05, *p* = 0.049, η_p_^2^ = 0.023), from baseline to post intervention. *Post-hoc* analyses revealed a greater improvement in the ACG compared with the IG (*p* = 0.022, η_p_^2^ = 0.028). This effect did not persist until follow-up, when no significant group differences were noted. PP analyses showed no significant between-group differences from pre to post in the overall model (*F*(2,159) = 2.99, *p* = 0.053, η_p_^2^ = 0.036).

The evaluation of the BDI-II showed a significant difference between groups from baseline to post assessment (*F*(2,259) = 3.23, *p* = 0.011, η_p_^2^ = 0.039) owing to the greater improvement in the IG compared to the WCG (*p* = 0.011, η_p_^2^ = 0.039). From baseline to follow-up, group differences were again significant (*F*(2,259) = 3.75, *p* = 0.025, η_p_^2^ = 0.028), with the IG improving significantly compared to the ACG (*p* = 0.011, η_p_^2^ = 0.034).

PP analyses corroborated these findings (*F*(2,159) = 3.63, *p* = 0.029, η_p_^2^ = 0.044), with the IG showing greater improvement in depressive symptoms than both the WCG and ACG However, this improvement was not significant from baseline to follow-up.

Significant group differences were found for the somatic-affective subscale of the BDI-II (*F*(2,259) = 3.12, *p* = 0.046, η_p_^2^ = 0.024) from baseline to follow-up. The IG showed significant improvement in depressive symptoms compared to the WCG (*p* = 0.015, η_p_^2^ = 0.035). The effect for group differences were significant baseline to follow-up (*F* (2,259) = 3.45, *p* = 0.032, η_p_^2^ = 0.026), with the IG showing significant improvement compared to the WCG (*p* = 0.050, η_p_^2^ = 0.023) and to the ACG (*p* = 0.018, η_p_^2^ = 0.030). For the cognitive subscale of the BDI-II, there were no significant group differences at any assessment point (all *p*s >.050).

PP analyses also showed a significant group differences for the somatic-affective subscale of the BDI-II from baseline to post assessment (*F*(2,159) = 3.91, *p* = 0.022, η_p_^2^ = 0.047). A significant decrease in somatic-affective depressive symptoms was found in the IG compared to the WCG (*p* = .015, η_p_^2^ = .052), and the ACG also improved compared to the WCG (*p* = 0.022, η_p_^2^ = 0.052). However, no significant differences were found between the groups from baseline to follow up. Similar to the ITT analyses, no group differences were found in the analysis of the cognitive subscale, neither from baseline to post nor from baseline to follow-up assessment.

Furthermore, the ITT analyses showed a significant improvement in pain catastrophizing as measured by the PCS (*F*(2,259) = 3.94, *p* = 0.021, η_p_^2^ = 0.030). The ACG improved relative to the WCG at post intervention (*p* = 0.006, η_p_^2^ = 0.045), however the IG failed to reach significance with the WCG (*p* = 0.052, η_p_^2^ = 0.022). Baseline to follow-up analysis showed no significant group differences for this outcome. PP analyses revealed no significant group differences on PCS scores from baseline to post or baseline to follow-up.

### Within-group differences

Results of the within-group differences for complete cases are shown in **Supplementary Table A** in the **Supplementary File 1**. Significant improvements emerged for all three groups (i.e., IG, WCG, ACG) from baseline to post and baseline to follow-up for BDI-II total, BDI-II Cognitive Subscale, BDI-II Somatic-Affective Subscale, QoL, and PHQ-9. For the primary outcome, the von Korff Disability Score, dependent sample *t*-tests showed significant reductions from baseline to post or baseline to follow-up within the WCG or the ACG (see Appendix). However, for the IG it was only significant from baseline to follow-up (*t*(59) = 2.492, *p* = 0.016, *d* = 0.322).

### Client satisfaction questionnaire-8 and subjective appraisal

#### CSQ-8

In total, 45 participants at post and 38 participants at follow-up completed the subjective evaluation of Lenio (CSQ-8; **Table 2**). Participants’ subjective appraisal of COGITO was not assessed. The great majority of the participants assessed the program’s quality positively (post: 91.1%; follow-up: 79.0%) and reported experiencing relief from depressive symptoms (post: 80.0%). Approximately two out of three participants indicated that they received the help they expected (post: 66.7%; follow-up: 65.8%), that their needs were met (post: 62.2%; follow-up: 63.1%), and that they were satisfied with the assistance they received (post: 72.9%; follow-up: 68.4%). Lenio enabled participants to better cope with their problems (post: 66.7%; follow-up: 76.4%). Overall, approximately three out of four participants expressed satisfaction with the program (post: 75.6%; follow-up: 73.6%). They expressed willingness to use Lenio again (post: 66.7%; follow-up: 68.4%) and to recommend it to others (post: 66.7%; follow-up: 73.7%).

**Table 2:** Subjective appraisal of participants who used Lenio (questionnaire adapted from the CSQ-8).

Item	Lenio post (*n* = 45)	Lenio follow-up (*n* = 38)
How do you rate the quality of Lenio? (not good (1), less good (2) vs. good (3), excellent (4)) (recoded)	3.04 (0.47) [91.1%]	3.00 (0.74) [79.0%]
Did you receive the type of treatment you expected to receive? (not at all (1), not really (2) vs. in general yes (3), yes absolutely (4))	2.62 (0.77) [66.7%]	2.71 (0.80) [65.8%]
To which extent did Lenio meet your needs? (it did not meet my needs (1), it met few of my needs (2) vs. it met most of my needs (3), it met all of my needs (4)) (recoded)	2.60 (0.81) [62.2%]	2.63 (0.82) [63.1%]
Would you recommend the manual to a friend with similar symptoms? (definitely not (1), probably not (2) vs. probably yes (3), absolutely (4))	3.18 (0.77) [66.7%]	3.08 (0.91) [73.7%]
How happy are you about the extent of the help you have received through using the program? (dissatisfied (1), somewhat dissatisfied (2) vs. mostly satisfied (3), very satisfied (4))	2.76 (0.71) [72.9%]	2.92 (0.94) [68.4%]
Did Lenio help you cope with your problems more successfully? (no, it did not help me at all (1), no, it did not help me that much (2) vs. yes, it helped me a little (3), yes, it absolutely helped me (4)) (recoded)	2.84 (0.71) [66.7%]	2.97 (0.68) [76.4%]
How satisfied are you with Lenio in general? (very satisfied (1), mostly satisfied (2) vs. somewhat unsatisfied (3), unsatisfied (4))	2.91 (0.70) [75.6%]	2.95 (0.90) [73.6%]
Would you use Lenio again? (definitely not (1), probably not (2) vs. probably yes (3), yes (4))	2.93 (0.03) [66.7%]	2.97 (0.92) [68.4%]
Has Lenio reduced your pain symptoms? (added item)	2.30 (0.95) [40.0%]	
Has Lenio improved your well-being? (added item)	2.90 (0.88) [80.0%]	

Mean scores and standard deviations in parentheses; percentages in square brackets include the two positive response options; Lenio post = Post scores 8 weeks after baseline; Lenio follow-up = Follow-up scores 16 weeks after baseline.

#### Subjective appraisal

The subjective appraisal of the intervention, as assessed in a questionnaire separate from the CSQ-8, is presented in **Table 3**. Overall, the intervention received a positive evaluation at post (*n* = 43) and follow-up (*n* = 36). More than four out of five participants (post: 86.0%; follow-up: 83.3%) regarded the program as suitable for self-application and found its contents comprehensible (post: 93.1%; follow-up: 88.5%). A majority considered the program to be useful (post: 69.8%; 73.5%). In accordance with our previous findings, participants subjectively reported a reduction in their depressive symptoms after using Lenio (post: 56.2%; follow-up: 36.4%). However, only 25.7% at post and 36.4% at follow-up reported a reduction in pain symptoms as a result of using Lenio. A substantial proportion (post: 55.8%, follow-up: 62.1%) indicated they had difficulty motivating themselves to engage with the Internet-based intervention. Mostly, people were able to integrate Lenio well into their everyday life (post: 61.9%; follow-up: 61.1%).

**Table 3:** Subjective appraisal of participants who who used Lenio (measured with the self-developed questionnaire on the differential effectiveness of the three techniques).

Item	Lenio post (*n* = 43)	Lenio follow-up (*n* = 36)
I think Lenio is good for self-help and self-guidance.	3.35 (0.78) [86.1%]	3.11 (0.89) [83.3%]
I think Lenio is useful.	2.95 (0.92) [69.8%]	3.06 (1.04) [73.5%]
I found Lenio easy to understand.	3.48 (0.63) [93.1%]	3.34 (0.84) [88.5%]
I was able to integrate Lenio and the associated exercises well into my everyday life.	2.74 (1.01) [61.9%]	2.67 (0.90) [61.1%]
I had to force myself to use Lenio.	2.51 (1.10) [55.8%]	2.76 (1.12) [62.1%]
I feel that Lenio has reduced my pain.	1.89 (0.93) [25.7%]	2.12 (0.99) [36.4%]
I feel that Lenio has alleviated my emotional problems.	2.49 (1.04) [56.2%]	2.37 (1.00) [46.5%]

Mean scores and standard deviations in parentheses (4-point Likert scale: 1 = “not at all true,” 2 = “a little,” 3 = “mostly agree,” 4 = “absolutely”); percentages in square brackets include response options from “a little” to “absolutely”.

### Moderator and prediction analyses

In the moderator and predictor analyses, data from the PP group were used. Positive beta coefficients indicated that higher values of the moderator led to increased reduction of symptoms in the IG. The different standard deviations illustrate the differences between the two groups in terms of change scores from baseline to post assessments at different levels of the moderator: low (−1 SD), average (0), or high (+1 SD) treatment effect.

A significant moderation emerged from baseline to post for age. Older participants in the IG benefited significantly more compared to older participants in the WCG (*B* = 0.102, *SE* = 0.050, *t* = 2.042, *p* = .044, LLCI = 0.003, ULCI = 0.201). Moreover, higher WSQ scores, indicating the presence of multiple diagnoses, led to a greater reduction in depressive symptoms in the IG compared to the WCG (*B* = 1.41, *SE* = 0.677, *t* = 2.077, *p* = .040, LLCI = 0.065, ULCI = 2.747).

## Discussion

Our study set out to investigate whether a newly developed online self-help program (Lenio) in combination with a self-help smartphone app (COGITO) would reduce both pain and depression in a large and heterogeneous sample of individuals. Our hypotheses were only partially confirmed. The results demonstrate that Lenio/COGITO ameliorated pain and most secondary outcomes across time. Yet, these improvements were rarely larger than in the ACG and WCG, which unexpectedly improved as well. For pain interference reduction, the ACG even outperformed Lenio/COGITO from baseline to post intervention. However, this effect did not persist until follow-up. For depressive symptoms (measured by the BDI-II), Lenio/COGITO was significantly more effective than the WCG from baseline to post intervention. Additionally, the IG showed a sustained advantage over the ACG until follow-up. This effect was particularly pronounced for the somatic-affective subscale of the BDI-II, whereas no significant group differences were found for the cognitive subscale. We plan to return to this result in the future and consider the hypothesis that Lenio/COGITO is more effective for psychosomatic symptoms than for pain, which is different than we had initially intended.

### Somatic components of depression and chronic pain

Between-group differences for depression in favor of Lenio/COGITO were especially marked for the somatic-affective component of depression, decreasing significantly from baseline to post intervention and until follow-up in comparison to the WCG. Moreover, somatic-affective depression decreased in the IG significantly from baseline to follow-up in comparison to the ACG. The reduction of somatic-affective depression symptoms, such as fatigue, disruptions in sleep patterns, and a sense of exhaustion (49), in patients with chronic pain could have significant implications for the long-term management of pain intensity and pain interference. Alleviating somatic-affective symptoms through targeted interventions such as Lenio/COGITO might break the vicious circle of pain and depression reinforcement.

### Active control group

Individuals who received access to the smartphone app MCT & More (ACG condition) achieved a significantly stronger reduction in pain symptoms compared to the Lenio*/*COGITO group to post intervention, even though this intervention was not aimed at reducing chronic pain and contained only a subset of exercises conveyed by Lenio/COGITO. Thus, MCT & More did not contain any contents beyond those conveyed by the experimental condition. Importantly, the IG used the MCT & More app more frequently than Lenio/COGITO. The main reason for this could be that it is easier to integrate into everyday life. As discussed in the introduction, low-threshold interventions are becoming increasingly popular in the field of mental health. Smartphones in particular, which are regularly used in daily life, are outperforming Internet-based programs such as Lenio. Recent research (50) tentatively suggests that the combination of different media and conveying multiple techniques, in our case the combination of an Internet-based platform and a smartphone app, may overwhelm and confuse many participants leading to decreased adherence and less reduction in symptoms. A more focused approach, as in the ACG of the current study, may thus be advantageous (“less is more”). Still, the greater reduction of pain symptoms in the ACG points to the need for a revision of the Lenio/COGITO program, possibly by providing patients with more focused guidance.

### Wait-list control group

While previous research suggests that waitlist control groups typically inflate effect sizes in favor of the experimental intervention (51, 52), this unlikely applies to our study, as the WCG also demonstrated notable improvements. Several factors could explain this unexpected result. First, natural symptom fluctuations or regression to the mean may have contributed to improvements in the WCG. However, Sean and colleagues (53) argue that symptom improvements in untreated chronic pain populations cannot be entirely attributed to regression to the mean alone. Their study suggests an ‘Effect of Care,’ where participants benefit simply from study participation, potentially due to increased self-reflection, structured symptom tracking, and interactions with study staff. Second, participants might have sought alternative treatments or coping strategies while waiting for the intervention, influencing their symptom trajectories. Future research should further explore the conditions under which WCGs improve and refine study designs to account for such influences.

### Long-term effects of cognitive behavioral therapy on chronic pain via a reduction in depression

According to prior research (54), reducing depressive symptoms in patients with chronic pain promises a long-term reduction in pain symptoms. Pain in turn impacts depression (55), speaking for a bidirectional relationship between the two conditions. Although Lenio/COGITO did not lead to significant reductions in pain interference and severity compared to the ACG and WCG in a period of 16 weeks, considering the significant reduction in depressive symptoms in the IG compared to the other groups through follow-up, longer observation intervals of the Lenio/COGITO intervention might reveal an improvement in overall pain symptoms due to enhanced mental health and coping mechanisms. Also, considering that in the subjective evaluation of the program the perceived pain reduction significantly increased from the post to the follow-up evaluation, one might hypothesize that the observation period was too short to accurately capture any improvement in pain interference (see **Table 3**). This can be viewed as mere speculation due to a lack of further long-term assessments in our study, but there is some evidence in favor of this hypothesis. Samwel et al. (56) found a reduction in pain after 12 months following CBT that included stress management, problem solving, and relaxation. Likewise, Zanini and colleagues (57) assume that effects in pain reduction only manifest after months. Such “sleeper effects” are well known for other disorders, too (58). To illustrate, Moritz and colleagues examined the effects of metacognitive training (MCT) in patients with schizophrenia and found significant improvements on some outcomes, but only when measured three years after initial assessment (58). These findings further stress the need for long-term investigation of Lenio/COGITO. However, these findings also raise the question of whether pain-specific psychotherapy is needed or whether treatment of depressive symptoms alone may also lead to “sleeper effects” on pain symptoms.

### Age-related differences

Moderation analyses indicate that Lenio/COGITO resulted in a stronger reduction in depressive symptoms among older participants during the pre-to-post interval compared to the WCG. This is in line with existing research showing higher adherence to Internet-based self-help programs with higher age (59). This may have to do with less experience with such programs in this age group, leading to higher treatment expectations and enthusiasm than in younger patients, who are more Internet-savvy and perhaps find the design and presentation less appealing. In line with this, many young individuals do not complete Internet-based programs, and their adherence (e.g., frequency of usage) to such programs is often low (60).

Achilles et al. (61) discuss limited time, technical difficulties, repetitive content, concerns about privacy and anonymity, and other factors as challenges that lead to reduced involvement and participation in Internet-based interventions by young people. While their results were collected in a sample younger than ours (12–25 years), we expect that further program personalization, gamification elements, participatory design, and use supported by a therapist could contribute to enhancing young people’s compliance with programs such as Lenio/COGITO. To conclude, recognizing the interplay of age-related preferences, technological familiarity, and intricacies in program design can help in developing tailored strategies to boost involvement among diverse age groups in Internet-based self-help programs such as Lenio.

### The biopsychosocial model and heterogeneity in pain

The biopsychosocial model of chronic pain acknowledges the complex interplay between psychological, physical, and social factors in pain management (62). For example, individuals with pain arising from accidents or from lifelong chronic conditions (e.g., rheumatoid arthritis) may have very different treatment needs, ranging from strictly medical to psychological interventions (63, 64).

In developing Lenio/COGITO, we did not differentiate between the various causes of pain; we aimed instead for a broadly applicable program. This approach has both advantages and disadvantages. It might be beneficial to introduce additional program packages for other causes of pain not yet mentioned, such as cancer, spinal problems, operations and injuries, or rheumatoid arthritis (65), to create a more customizable approach.

### Outlook

Acknowledging the effects on somatic components of depression and in line with our moderation analysis, Lenio in combination with COGITO could be especially useful in the treatment of somatic depression, particularly in older patients. Considering that the subjective feedback on the Lenio program was generally positive, the integration of Lenio into existing rehabilitation approaches for pain should be considered when the program is revised as outlined above (more focus and guidance). Lenio could serve to bridge waiting times, providing affected individuals with initial self-help tools and psychoeducation. It is yet to be tested whether utilizing Lenio in a preventive fashion could ward off the onset of depression and perhaps also pain and whether the positive impact of using Lenio in combination with COGITO, especially in reducing depressive symptoms, could be leveraged to protect individuals from the emergence of depressive symptoms through regular participation in interactive exercises such as those available in the COGITO app.

### Limitations

Strengths of this study include the large sample size and the inclusion of an ACG as well as a WCG. Several limitations should be acknowledged. First, the study’s reliance on self-reported assessments, necessitated by data protection constraints, led to some uncertainty regarding the precision and dependability of the gathered data. Another key limitation is the participants’ relatively low (self-reported) engagement with the program.

Moreover, the observed improvements in the WCG may reflect factors such as increased self-monitoring due to study participation, anticipation effects from awaiting the intervention, or natural symptom fluctuations. These effects, while beyond the scope of the current study, highlight the potential influence of external variables in interpreting control group results and underscore the need for careful consideration in future research.

Additionally, the study faced challenges with moderate re-assessment rates, which could potentially impact the broader applicability of the findings. Moreover, the comparatively brief duration of the follow-up period restricts our ability to assess long-term effects, underscoring the need for further research. Our hypothesis that a reduction in depression may reduce pain at a later point in time also should be tested.

## Conclusion

In conclusion, this study found that pain interference and pain intensity were reduced using the Internet-based intervention Lenio and its accompanying self-help app COGITO, but not significantly larger in comparison to the ACG and WCG. However, Lenio/COGITO was especially successful for the treatment of somatic symptoms of depression, and this may be considered as the primary outcome in future studies. Lenio*/*COGITO might serve as a valuable bridge to conventional psychotherapy, psychoeducation, and overall improvement of well-being. Future research should focus on long-term effects of psychological self-help tools to measure gradual changes and consider the addition of specialized program packages to increase usage and accommodate the heterogeneity of chronic pain.

## Data Availability

The raw data supporting the conclusions of this article will be made available by the authors, without undue reservation.
