# Supplementary material for: Two in one: a randomized controlled trial on an internet-based intervention (Lenio) for management of both chronic pain and depressive symptoms
Source: Front Psychiatry. 2025 Mar 18;16:1528128. doi: 10.3389/fpsyt.2025.1528128 (PMC11959166; doi:10.3389/fpsyt.2025.1528128)
Supplement: Supplementary file 1 [file Supplementaryfile1.pdf]

Appendix

Table A. *Complete-case (CC), per-protocol (PP), and intention-to-treat (ITT) analyses across time for primary and secondary outcomes between and within groups from baseline to post intervention*

Baseline to Post Questionnaire Results	WLC			IG			ACG			ITT Baseline to Post (WLC: n =72, IG: n = 97, App: n = 94)	PP Baseline to Post: IG logged in at least once, usage of app (WLC: n = 53, IG: n = 62, App: n = 48)	CC Baseline to Post (WLC: n = 53, IG: n = 69, App: n = 70)
	Baseline	Post (n = 53)	Fu (n = 54)	Baseline	Post (n = 69)	Fu (n = 60)	Baseline	Post (n = 70)	Fu (n = 64)			
BDI-II Total Score	24.96 (9.36)	22.06 (10.59) [p = .008]	18.30 (10.44) [p < .001]	25.35 (10.63)	19.28 (11.64) [p < .001]	16.83 (11.32) [p < .001]	25.00 (9.95)	19.67 (12.05) [p < .001]	20.19 (12.35) [p < .001]	$F(2,259) = 3.23$, p = .041, $\eta_p^2 = .024$ [IG > WLC: p = .011, $\eta_p^2 = .039$; ACG = IG: p = .485, $\eta_p^2 = .003$; App ≥ WLC: p = .078, $\eta_p^2 = .019$]	$F(2,159) = 3.63$, p = .029, $\eta_p^2 = .044$ [IG > WLC: p = .017, $\eta_p^2 = .049$; IG = ACG: p = .792, $\eta_p^2 = .001$; ACG > WLC: p = .032, $\eta_p^2 = .046$]	$F(2,188) = 2.60$, p =.077, $\eta_p^2 = .027$ [IG > WLC: p = .025, $\eta_p^2 = .041$; IG = ACG: p = .584, $\eta_p^2 = .002$; ACG ≥ WLC: p = .096, $\eta_p^2 = .023$]
BDI-II Cognitive Subscale	9.00 (5.02)	(n = 53) 7.72 (5.41) [p = .015]	(n = 54) 6.02 (5.47) [p < .001]	8.65 (5.36)	(n = 69) 6.54 (4.86) [p < .001]	(n = 60) 5.50 (4.88) [p < .001]	8.64 (5.10)	(n = 70) 6.77 (5.56) [p < .001]	(n = 64) 6.95 (5.73) [p = .002]	$F(2,259) = 1.12$, p =.329, $\eta_p^2 = .009$ [IG = WLC: p = .130, $\eta_p^2 = .014$; ACG = IG: p = .823, $\eta_p^2 = .000$; App ≥ WLC: p = .272, $\eta_p^2 = .007$]	$F(2,159) = 0.64$, p = .526, $\eta_p^2 = .008$ [IG ≥ WLC: p = .088, $\eta_p^2 = .026$; IG = ACG: p = .908, $\eta_p^2 = .000$; ACG = WLC: p = .164, $\eta_p^2 = .020$]	$F(2,188) = 1.10$, p = .334, $\eta_p^2 = .012$ [IG = WLC: p = .122, $\eta_p^2 = .020$; IG = ACG: p = .672, $\eta_p^2 = .001$; ACG = WLC: p = .330, $\eta_p^2 = .008$]
BDI-II Somatic-Affective Subscale	15.96 (5.85)	(n = 53) 14.34 (6.46) [p = .021]	(n = 54) 12.28 (6.16) [p < .001]	16.70 (6.19)	(n = 69) 12.74 (7.67) [p < .001]	(n = 60) 11.33 (7.22) [p < .001]	16.33 (6.15)	(n = 70) 12.90 (7.53) [p < .001]	(n = 64) 13.23 (7.66) [p < .001]	$F(2,259) = 3.12$, p =.046, $\eta_p^2 = .024$ [IG > WLC: p = .015, $\eta_p^2 = .035$; ACG = IG: p = .574, $\eta_p^2 = .002$; App ≥ WLC: p = .057, $\eta_p^2 = .022$]	$F(2,159) = 3.91$, p =.022, $\eta_p^2 = .047$ [IG > WLC: p = .015, $\eta_p^2 = .052$; IG = ACG: p = .742, $\eta_p^2 = .001$; ACG > WLC: p = .022, $\eta_p^2 = .052$]	$F(2,188) = 2.98$, p = .053, $\eta_p^2 = .031$ [IG > WLC: p = .020, $\eta_p^2 = .045$; IG = ACG: p = .589, $\eta_p^2 = .002$; ACG ≥ WLC: p = .061, $\eta_p^2 = .029$]
Von Korff Disability Score	56.54 (20.50)	(n = 53) 52.20 (19.90)) [p = .033]	(n = 54) 46.98 (26.69) [p = .001]	54.00 (23.34)	(n = 69) 51.69 (22.89) [p = .119]	(n = 60) 46.67 (22.54) [p = .003]	60.24 (25.89)	(n = 70) 50.43 (23.07) [p < .001]	(n = 64) 52.50 (24.42) [p < .001]	$F(2,259) = 3.05$, p =.049, $\eta_p^2 = .023$ [IG = WLC: p = .869, $\eta_p^2 = .000$;	$F(2,159) = 2.99$, p = .053, $\eta_p^2 = .036$ [IG = WLC: p = .722, $\eta_p^2 = .001$;	$F(2,188) = 4.04$, p =.019, $\eta_p^2 = .041$ [IG = WLC: p = .509, $\eta_p^2 = .004$;

										ACG > IG: p =<br>.022, η_p^2 = .028;<br>ACG ≥ WLC: p =<br>.071, η_p^2 = .020]	ACG > IG: p =<br>.019, η_p^2 = .050;<br>ACG ≥ WLC: p =<br>.079, η_p^2 = .031]	ACG > IG: p =<br>.006, η_p^2 = .054;<br>ACG ≥ WLC: p =<br>.073, η_p^2 = .027]
Von Korff Pain Intensity	2.847<br>(1.10)	(n = 53)<br>2.74<br>(1.21)<br>[p = .164]	(n = 54)<br>2.52<br>(1.26)<br>[p = .050]	(n = 69)<br>2.88<br>(1.12)	(n = 69)<br>2.83<br>(1.18)<br>[p = .567]	(n = 60)<br>2.52<br>(1.17)<br>[p = .016]	(n = 71)<br>3.06<br>(1.09)	(n = 71)<br>2.70<br>(1.25)<br>[p = .003]	(n = 64)<br>2.90<br>(1.15)<br>[p = .011]	F(2,259) = 0.21, p = .207, η_p^2 = .012<br>[IG = WLC: p =<br>.702, η_p^2 = .001;<br>IG = ACG: p =<br>.113, η_p^2 = .013;<br>ACG = WLC: p =<br>.213, η_p^2 = .010]	F(2,159) = 1.57, p = .211, η_p^2 = .019<br>[IG = WLC: p =<br>.784, η_p^2 = .001;<br>IG = ACG: p =<br>.130, η_p^2 = .021;<br>ACG = WLC: p =<br>.169, η_p^2 = .019]	F(2,188) = 0.92, p =.237, η_p^2 = .015<br>[IG = WLC: p =<br>.579, η_p^2 = .003;<br>IG = ACG: p =<br>.122, η_p^2 = .018;<br>ACG = WLC: p =<br>.289, η_p^2 = .009]
IEQ Total Score	21.30<br>(10.12)	(n = 53)<br>20.89<br>(9.04)<br>[p = .628]	(n = 54)<br>19.80<br>(10.79)<br>[p = .104]	23.03<br>(9.40)	(n = 69)<br>21.38<br>(10.32)<br>[p = .062]	(n = 60)<br>19.17<br>(9.74)<br>[p < .001]	24.74<br>(10.84)	(n = 70)<br>21.91<br>(10.76)<br>[p < .001]	(n = 64)<br>21.97<br>(10.70)<br>[p < .001]	F(2,259) = 1.27, p = .282, η_p^2 = .010<br>[IG = WLC: p =<br>.320, η_p^2 = .006;<br>IG = ACG: p =<br>.510, η_p^2 = .002;<br>ACG ≥ WLC: p =<br>.094, η_p^2 = .017]	F(2,159) = 0.45, p = .636, η_p^2 = .006<br>[IG = WLC: p =<br>.446, η_p^2 = .005;<br>IG = ACG: p =<br>.990, η_p^2 = .000;<br>ACG = WLC: p =<br>.380, η_p^2 = .008]	F(2,188) = 1.12, p =.329, η_p^2 = .012<br>[IG = WLC: p =<br>.485, η_p^2 = .004;<br>IG = ACG: p =<br>.426, η_p^2 = .005;<br>ACG = WLC: p =<br>.113, η_p^2 = .021]
WHOQOL-BREF (Quality of life global item)	2.71 (0.72)	(n = 52)<br>2.94<br>(0.85)<br>[p = .027]	(n = 54)<br>3.11<br>(0.84)<br>[p = .005]	2.64<br>(0.73)	(n = 69)<br>2.94<br>(0.84)<br>[p = .001]	(n = 60)<br>2.98<br>(0.97)<br>[p = .002]	2.63<br>(0.77)	(n = 70)<br>2.94<br>(0.76)<br>[p < .001]	(n = 64)<br>2.88<br>(0.77)<br>[p = .012]	F(2,259) = 0.19, p = .831, η_p^2 = .001<br>[IG = WLC: p =<br>.638, η_p^2 = .001;<br>IG = ACG: p =<br>.940, η_p^2 = .000;<br>ACG = WLC: p =<br>.547, η_p^2 = .002]	F(2,158) = 0.06, p = .940, η_p^2 = .001<br>[IG = WLC: p =<br>.768, η_p^2 = .001;<br>IG = ACG: p =<br>.990, η_p^2 = .000;<br>ACG = WLC: p =<br>.760, η_p^2 = .001]	F(2,187) = 0.12, p =.889, η_p^2 = .001<br>[IG = WLC: p =<br>.712, η_p^2 = .001;<br>IG = ACG: p =<br>.951, η_p^2 = .000;<br>ACG = WLC: p =<br>.628, η_p^2 = .002]
PHQ-9	12.42 (4.67)	(n = 53)<br>10.78<br>(4.97)<br>[p = .002]	(n = 54)<br>9.96<br>(4.87)<br>[p < .001]	13.52<br>(5.82)	(n = 69)<br>11.06<br>(5.59)<br>[p < .001]	(n = 60)<br>10.05<br>(5.81)<br>[p < .001]	13.36<br>(5.16)	(n = 70)<br>11.44<br>(6.19)<br>[p < .001]	(n = 64)<br>11.49<br>(6.10)<br>[p < .001]	F(2,259) = 1.33, p = .265, η_p^2 = .010<br>[IG = WLC: p =<br>.152, η_p^2 = .012;<br>IG = ACG: p =<br>.196, η_p^2 = .009;<br>ACG = WLC: p =<br>.763, η_p^2 = .001]	F(2,159) = 0.68, p = .508, η_p^2 = .008<br>[IG = WLC: p =<br>.272, η_p^2 = .011;<br>IG = ACG: p =<br>.445, η_p^2 = .005;<br>ACG = WLC: p =<br>.731, η_p^2 = .001]	F(2,188) = 0.52, p =.599, η_p^2 = .005<br>[IG = WLC: p =<br>.404, η_p^2 = .006;<br>IG = ACG: p =<br>.416, η_p^2 = .005;<br>ACG = WLC: p =<br>.833, η_p^2 = .000]

PCS Total Score	22.70 (9.90)	(*n* = 53)<br>22.66<br>(10.02)<br>[*p* = .975]	(*n* = 54)<br>20.59<br>(10.54)<br>[*p* = .150]	24.39<br>(9.45)	(*n* = 69)<br>21.68<br>(10.42)<br>[*p* = .008]	(*n* = 60)<br>19.80<br>(10.59)<br>[*p* < .001]	25.10<br>(11.92)	(*n* = 70)<br>20.61<br>(12.27)<br>[*p* < .001]	(*n* = 64)<br>21.47<br>(11.52)<br>[*p* < .001]	$F(2,259) = 3.94$, $p = .021$, $\eta_p^2 = .030$<br>[IG ≥ WLC: $p = .052$, $\eta_p^2 = .022$;<br>IG = ACG: $p = .384$, $\eta_p^2 = .004$;<br>ACG > WLC: $p = .006$, $\eta_p^2 = .045$]	$F(2,159) = 2.71$, $p = .069$, $\eta_p^2 = .033$<br>[IG = WLC: $p = .125$, $\eta_p^2 = .021$;<br>IG = ACG: $p = .496$, $\eta_p^2 = .004$;<br>ACG > WLC: $p = .024$, $\eta_p^2 = .051$]	$F(2,188) = 3.77$, $p = .025$, $\eta_p^2 = .039$<br>[IG = WLC: $p = .139$, $\eta_p^2 = .018$;<br>IG = ACG: $p = .209$, $\eta_p^2 = .012$;<br>ACG > WLC: $p = .006$, $\eta_p^2 = .061$]
FABQ1 Total Score	9.29 (5.84)	(*n* = 52)<br>10.58<br>(5.85)<br>[*p* =.053]	(*n* = 54)<br>10.30<br>(7.18)<br>[*p* = .263]	11.31<br>(5.82)	(*n* = 69)<br>11.28<br>(5.40)<br>[*p* = .945]	(*n* = 60)<br>11.65<br>(6.12)<br>[*p* = .461]	11.19<br>(6.53)	(*n* = 70)<br>10.31<br>(6.58)<br>[*p* = .163]	(*n* = 64)<br>10.61<br>(6.52)<br>[*p* = .209]	$F(2,259) = 2.18$, $p = .115$. $\eta_p^2 = .017$<br>[IG = WLC: $p = .271$. $\eta_p^2 = .007$;<br>IG = ACG: $p = .289$. $\eta_p^2 = .006$;<br>ACG > WLC: $p = .037$. $\eta_p^2 = .026$]	$F(2.158) = 1.17$, $p = .313$, $\eta_p^2 = .015$<br>[IG = WLC: $p = .315$, $\eta_p^2 = .009$;<br>IG = ACG: $p = .594$, $\eta_p^2 = .003$;<br>ACG = WLC: $p = .116$, $\eta_p^2 = .025$]	$F(2,187) = 1.61$, $p = .203$, $\eta_p^2 = .017$<br>[IG = WLC: $p = .533$, $\eta_p^2 = .003$;<br>IG = ACG: $p = .267$, $\eta_p^2 = .009$;<br>ACG ≥ WLC: $p = .068$, $\eta_p^2 = .028$]
FABQ2 Total Score	15.15<br>(10.97)	(*n* = 52)<br>18.81<br>(11.89)<br>[*p* < .001]	(*n* = 54)<br>16.17<br>(12.85)<br>[*p* = .221]	14.97<br>(11.00)	(*n* = 69)<br>17.55<br>(10.42)<br>[*p* = .013]	(*n* = 60)<br>14.82<br>(11.11)<br>[*p* = .807]	14.91<br>(11.38)	(*n* = 70)<br>16.14<br>(12.39)<br>[*p* = .301]	(*n* = 64)<br>13.90<br>(10.89)<br>[*p* = .408]	$F(2,259) = 1.70$, $p = .186$, $\eta_p^2 = .013$<br>[IG = WLC: $p = .237$, $\eta_p^2 = .008$;<br>IG = ACG: $p = .425$, $\eta_p^2 = .003$;<br>ACG = WLC: $p = .085$, $\eta_p^2 = .018$]	$F(2,158) = 2.55$, $p = .082$, $\eta_p^2 = .031$<br>[IG = WLC: $p = .134$, $\eta_p^2 = .020$;<br>IG = ACG: $p = .337$, $\eta_p^2 = .009$;<br>ACG > WLC: $p = .037$, $\eta_p^2 = .044$]	$F(2,187) = 1.43$, $p = .243$, $\eta_p^2 = .015$<br>[IG = WLC: $p = .408$, $\eta_p^2 = .006$;<br>IG = ACG: $p = .345$, $\eta_p^2 = .007$;<br>ACG = WLC: $p = .111$, $\eta_p^2 = .021$]
FSS<br>Catastrophizing	15.15<br>(10.97)	(*n* = 52)<br>18.81<br>(11.89)<br>[*p* < .001]	(*n* = 54)<br>16.17<br>(12.85)<br>[*p* = .221]	14.97<br>(11.00)	(*n* = 69)<br>17.55<br>(10.42)<br>[*p* = .013]	(*n* = 60)<br>14.82<br>(11.11)<br>[*p* = .807]	14.91<br>(11.38)	(*n* = 70)<br>16.14<br>(12.39)<br>[*p* = .301]	(*n* = 64)<br>13.90<br>(10.89)<br>[*p* = .408]	$F(2,259) = 1.10$, $p = .334$, $\eta_p^2 = .008$<br>[IG = WLC: $p = .161$, $\eta_p^2 = .012$;<br>IG = ACG: $p = .595$, $\eta_p^2 = .002$;<br>ACG = WLC: $p = .312$, $\eta_p^2 = .006$]	$F(2,158) = 1.17$, $p = .313$, $\eta_p^2 = .015$<br>[IG = WLC: $p = .197$, $\eta_p^2 = .015$;<br>IG = ACG: $p = .714$, $\eta_p^2 = .001$;<br>ACG = WLC: $p = .319$, $\eta_p^2 = .010$]	$F(2,187) = 0.82$, $p = .441$, $\eta_p^2 = .009$<br>[IG = WLC: $p = .264$, $\eta_p^2 = .011$;<br>IG = ACG: $p = .803$, $\eta_p^2 = .000$;<br>ACG = WLC: $p = .290$, $\eta_p^2 = .009$]
FSS<br>Active Coping	15.15<br>(10.97)	(*n* = 52)<br>18.81<br>(11.89)<br>[*p* < .001]	(*n* = 54)<br>16.17<br>(12.85)<br>[*p* = .221]	14.97<br>(11.00)	(*n* = 69)<br>17.55<br>(10.42)<br>[*p* = .013]	(*n* = 60)<br>14.82<br>(11.11)<br>[*p* = .807]	14.91<br>(11.38)	(*n* = 70)<br>16.14<br>(12.39)<br>[*p* = .301]	(*n* = 64)<br>13.90<br>(10.89)<br>[*p* = .408]	$F(2,259) = 0.16$, $p = .855$, $\eta_p^2 = .001$<br>[IG = WLC: $p = .624$, $\eta_p^2 = .001$;	$F(2,158) = .64$, $p = .526$, $\eta_p^2 = .008$<br>[IG = WLC: $p = .624$, $\eta_p^2 = .002$;	$F(2,187) = .21$, $p = .811$, $\eta_p^2 = .002$<br>[IG = WLC: $p = .845$, $\eta_p^2 = .000$;

	IG = ACG: p = .917, η_p^2 = .000; ACG = WLC: p = .649, η_p^2 = .001]	IG = ACG: p = .519, η_p^2 = .004; ACG = WLC: p = .253, η_p^2 = .013]	IG = ACG: p = .684, η_p^2 = .001; ACG = WLC: p = .517, η_p^2 = .004]

Note. WLC = Waitlist-control group; IG = Intervention group; ACG = Active-control group; Baseline = Baseline assessment; Post = Post Assessment; FU = Follow-up assessment; BDI – II = Beck Depression Inventory; PHQ-9 = Patient Health Questionnaire; PCS Patient Catastrophizing Scale; FABQ = Fear Avoidance Beliefs Questionnaire; FSS = Pain Self-Efficacy Questionnaire; ITT = Intention to treat; PP = Per protocol, CC = Complete cases.

Table B. *Complete-case (CC), per-protocol (PP), and intention-to-treat (ITT) analyses across time for primary and secondary outcomes between and within groups from baseline to follow-up intervention*

Baseline to Follow-Up Questionnaire Results	WLC			IG			ACG			ITT Baseline to FU (WLC: n = 72, IG: n = 97, ACG: n = 94)	PP Baseline to FU: IG logged in at least once, usage of ACG (WLC: n =51, IG: n = 54 , ACG: n = 44)	CC: Baseline to FU (WLC: n = 54, IG: n = 60 , ACG: n = 64)
	Baseline	Post (n = 53)	FU (n = 54)	Baseline	Post (n = 69)	FU (n = 60)	Baseline	Post (n = 70)	FU (n = 64)			
BDI-II Total Score	24.96 (9.36)	22.06 (10.59) [p = .008]	18.30 (10.44) [p < .001]	25.35 (10.63)	19.28 (11.64) [p < .001]	16.83 (11.32) [p < .001]	25.00 (9.95)	19.67 (12.05) [p < .001]	20.19 (12.35) [p < .001]	F(2,259) = 3.75, p = .025, η_p^2 = .028 [IG ≥ WLC: p = .072, η_p^2 = .019; IG > ACG: p = .011, η_p^2 = .034; ACG = WLC: p = .452, η_p^2 = .003]	F(2,145) = 1.41, p = .248, η_p^2 = .019 [IG = WLC: p = .199, η_p^2 = .016; IG = ACG: p = .146, η_p^2 = .022; ACG = WLC: p = .803, η_p^2 = .001]	F(2,174) = 3.90, p = .022, η_p^2 = .043 [IG = WLC: p = .123, η_p^2 = .021; IG > ACG: p = .010, η_p^2 = .054; ACG = WLC: p = .234, η_p^2 = .012]
BDI-II Cognitive Subscale	9.00 (5.02)	(n = 53) 7.72 (5.41) [p = .015]	(n = 54) 6.02 (5.47) [p < .001]	8.65 (5.36)	(n = 69) 6.54 (4.86) [p < .001]	(n = 60) 5.50 (4.88) [p < .001]	8.64 (5.10)	(n = 70) 6.77 (5.56) [p < .001]	(n = 64) 6.95 (5.73) [p = .002]	F(2,259) = 1.96, p = .143, η_p^2 = .015 [IG = WLC: p = .389, η_p^2 = .004; IG ≥ ACG: p = .051, η_p^2 = .020; ACG = WLC: p = .314, η_p^2 = .006]	F(2,145) = 0.81, p =.447, η_p^2 = .011 [IG = WLC: p = .506, η_p^2 = .004; IG = ACG: p = .220, η_p^2 = .016; ACG = WLC: p = .517, η_p^2 = .005]	F(2,174) = 2.95, p = .055, η_p^2 = .033 [IG = WLC: p = .326, η_p^2 = .009; IG > ACG: p = .021, η_p^2 = .043; ACG = WLC: p = .173, η_p^2 = .016]
BDI-II Somatic-Affective Subscale	15.96 (5.85)	(n = 53) 14.34 (6.46) [p = .021]	(n = 54) 12.28 (6.16) [p < .001]	16.70 (6.19)	(n = 69) 12.74 (7.67) [p < .001]	(n = 60) 11.33 (7.22) [p < .001]	16.33 (6.15)	(n = 70) 12.90 (7.53) [p < .001]	(n = 64) 13.23 (7.66) [p < .001]	F(2,259) = 3.45, p = .032, η_p^2 = .026 [IG > WLC: p = .050, η_p^2 = .023; IG > ACG: p = .018, η_p^2 = .030; ACG ≥ WLC: p = .057, η_p^2 = .022]	F(2,145) = 1.64, p =.197, η_p^2 = .022 [IG = WLC: p = .112, η_p^2 = .025; IG = ACG: p = .164, η_p^2 = .020; ACG = WLC: p = .913, η_p^2 = .000]	F(2,174) = 3.64, p = .028, η_p^2 = .040 [IG ≥ WLC: p = .083, η_p^2 = .027; IG > ACG: p = .014, η_p^2 = .049; ACG = WLC: p = .379, η_p^2 = .007]
Von Korff Disability Score	56.54 (20.50)	(n = 53) 52.20 (19.90)) [p = .033]	(n = 54) 46.98 (26.69) [p = .001]	54.00 (23.34)	(n = 69) 51.69 (22.89) [p = .119]	(n = 60) 46.67 (22.54) [p = .003]	60.24 (25.89)	(n = 70) 50.43 (23.07) [p < .001]	(n = 64) 52.50 (24.42) [p < .001]	F(2,259) = 0.03, p =.968, η_p^2 = .000 [IG = WLC: p = .807, η_p^2 = .000;	F(2,145) = 0.34, p = .711, η_p^2 = .005 [IG = WLC: p = .760, η_p^2 = .001;	F(2,174) = 0.23, p = .794, η_p^2 = .003 [IG = WLC: p = .557, η_p^2 = .003;

										IG = ACG: p = .973, η_p^2 = .000;<br>ACG = WLC: p = .836, η_p^2 = .000]	IG = ACG: p = .538, η_p^2 = .004;<br>ACG = WLC: p = .450, η_p^2 = .006]	IG = ACG: p = .884, η_p^2 = .000;<br>ACG = WLC: p = .587, η_p^2 = .003]
Von Korff Pain Intensity	2.847 (1.10)	(n = 53)<br>2.74 (1.21)<br>[p = .164]	(n = 54)<br>2.52 (1.26)<br>[p = .050]	(n = 69)<br>2.88 (1.12)	(n = 69)<br>2.83 (1.18)<br>[p = .567]	(n = 60)<br>2.52 (1.17)<br>[p = .016]	(n = 71)<br>3.06 (1.09)	(n = 71)<br>2.70 (1.25)<br>[p = .003]	(n = 64)<br>2.90 (1.15)<br>[p = .011]	F(2,259) = 0.52, p =.598, η_p^2 = .004<br>[IG = WLC: p = .648, η_p^2 = .001;<br>IG = ACG: p = .290, η_p^2 = .006;<br>ACG = WLC: p = .655, η_p^2 = .001]	F(2,145) = 0.23, p = .798, η_p^2 = .003<br>[IG = WLC: p = .792, η_p^2 = .001;<br>IG = ACG: p = .573, η_p^2 = .003;<br>ACG = WLC: p = .707, η_p^2 = .002]	F(2,174) = 0.32, p = .730, η_p^2 = .004<br>[IG = WLC: p = .835, η_p^2 = .000;<br>IG = ACG: p = .420, η_p^2 = .005;<br>ACG = WLC: p = .661, η_p^2 = .002]
IEQ Total Score	21.30 (10.12)	(n = 53)<br>20.89 (9.04)<br>[p = .628]	(n = 54)<br>19.80 (10.79)<br>[p = .104]	23.03 (9.40)	(n = 69)<br>21.38 (10.32)<br>[p = .062]	(n = 60)<br>19.17 (9.74)<br>[p < .001]	24.74 (10.84)	(n = 70)<br>21.91 (10.76)<br>[p < .001]	(n = 64)<br>21.97 (10.70)<br>[p < .001]	F(2,259) = 1.18, p = .309, η_p^2 = .009<br>[IG = WLC: p = .128, η_p^2 = .014;<br>IG = ACG: p = .401, η_p^2 = .004;<br>ACG = WLC: p = .479, η_p^2 = .003]	F(2,145) = 1.63, p = .199, η_p^2 = .022<br>[IG = WLC: p = .112, η_p^2 = .025;<br>IG = ACG: p = .149, η_p^2 = .022;<br>ACG = WLC: p = .831, η_p^2 = .001]	F(2,174) = 1.92, p = .149, η_p^2 = .022<br>[IG ≥ WLC: p = .060, η_p^2 = .032;<br>IG = ACG: p = .197, η_p^2 = .014;<br>ACG = WLC: p = .486, η_p^2 = .004]
WHOQOL-BREF (Quality of life global item)	2.71 (0.72)	(n = 52)<br>2.94 (0.85)<br>[p = .027]	(n = 54)<br>3.11 (0.84)<br>[p = .005]	2.64 (0.73)	(n = 69)<br>2.94 (0.84)<br>[p = .001]	(n = 60)<br>2.98 (0.97)<br>[p = .002]	2.63 (0.77)	(n = 70)<br>2.94 (0.76)<br>[p < .001]	(n = 64)<br>2.88 (0.77)<br>[p = .012]	F(2,259) = 0.86, p = .426, η_p^2 = .007<br>[IG = WLC: p = .855, η_p^2 = .000;<br>IG = ACG: p = .282, η_p^2 = .006;<br>ACG = WLC: p = .208, η_p^2 = .010]	F(2,145) = 0.72, p = .487, η_p^2 = .010<br>[IG = WLC: p = .898, η_p^2 = .000;<br>IG = ACG: p = .318, η_p^2 = .011;<br>ACG = WLC: p = .234, η_p^2 = .015]	F(2,174) = 0.84, p = .435, η_p^2 = .010<br>[IG = WLC: p = .782, η_p^2 = .001;<br>IG = ACG: p = .335, η_p^2 = .008;<br>ACG = WLC: p = .183, η_p^2 = .015]
PHQ-9	12.42 (4.67)	(n = 53)<br>10.78 (4.97)<br>[p = .002]	(n = 54)<br>9.96 (4.87)<br>[p < .001]	13.52 (5.82)	(n = 69)<br>11.06 (5.59)<br>[p < .001]	(n = 60)<br>10.05 (5.81)<br>[p < .001]	13.36 (5.16)	(n = 70)<br>11.44 (6.19)<br>[p < .001]	(n = 64)<br>11.49 (6.10)<br>[p < .001]	F(2,259) = 2.91, p = .056, η_p^2 = .022<br>[IG = WLC: p = .167, η_p^2 = .011;<br>IG > ACG: p = .022, η_p^2 = .028;<br>ACG = WLC: p = .530, η_p^2 = .002]	F(2,145) = 1.80, p = .169, η_p^2 = .024<br>[IG = WLC: p = .230, η_p^2 = .014;<br>IG ≥ ACG: p = .087, η_p^2 = .031;<br>ACG = WLC: p = .656, η_p^2 = .002]	F(2,174) = 2.64, p = .075, η_p^2 = .029<br>[IG = WLC: p = .219, η_p^2 = .014;<br>IG > ACG: p = .032, η_p^2 = .037;<br>ACG = WLC: p = .487, η_p^2 = .004]

PCS Total Score	22.70 (9.90)	(*n* = 53) 22.66 (10.02) [*p* = .975]	(*n* = 54) 20.59 (10.54) [*p* = .150]	24.39 (9.45)	(*n* = 69) 21.68 (10.42) [*p* = .008]	(*n* = 60) 19.80 (10.59) [*p* < .001]	25.10 (11.92)	(*n* = 70) 20.61 (12.27) [*p* < .001]	(*n* = 64) 21.47 (11.52) [*p* < .001]	$F(2,259) = 2.00$, $p = .139$, $\eta_p^2 = .015$ [IG ≥ WLC: $p = .052$, $\eta_p^2 = .023$; IG = ACG: $p = .366$, $\eta_p^2 = .004$; ACG = WLC: $p = .243$, $\eta_p^2 = .008$]	$F(2,145) = 1.41$, $p = .300$, $\eta_p^2 = .016$ [IG = WLC: $p = .152$, $\eta_p^2 = .020$; IG = ACG: $p = .426$, $\eta_p^2 = .007$; ACG = WLC: $p = .459$, $\eta_p^2 = .006$]	$F(2,174) = 1.43$, $p = .243$, $\eta_p^2 = .016$ [IG = WLC: $p = .116$, $\eta_p^2 = .022$; IG = ACG: $p = .506$, $\eta_p^2 = .004$; ACG = WLC: $p = .268$, $\eta_p^2 = .011$]
FABQ1 Total Score	9.29 (5.84)	(*n* = 52) 10.58 (5.85) [*p* =.053]	(*n* = 54) 10.30 (7.18) [*p* = .263]	11.31 (5.82)	(*n* = 69) 11.28 (5.40) [*p* = .945]	(*n* = 60) 11.65 (6.12) [*p* = .461]	11.19 (6.53)	(*n* = 70) 10.31 (6.58) [*p* = .163]	(*n* = 64) 10.61 (6.52) [*p* = .209]	$F(2,259) = 1.10$, $p = .338$, $\eta_p^2 = .008$ [IG = WLC: $p = .753$, $\eta_p^2 = .001$; IG = ACG: $p = .244$, $\eta_p^2 = .007$; ACG = WLC: $p = .166$, $\eta_p^2 = .012$]	$F(2,145) = 1.22$, $p = .299$, $\eta_p^2 = .017$ [IG = WLC: $p = .883$, $\eta_p^2 = .000$; IG = ACG: $p = .177$, $\eta_p^2 = .019$; ACG = WLC: $p = .151$, $\eta_p^2 = .022$]	$F(2,174) = 1.16$, $p = .315$, $\eta_p^2 = .013$ [IG = WLC: $p = .928$, $\eta_p^2 = .000$; IG = ACG: $p = .156$, $\eta_p^2 = .017$; ACG = WLC: $p = .205$, $\eta_p^2 = .014$]
FABQ2 Total Score	15.15 (10.97)	(*n* = 52) 18.81 (11.89) [*p* < .001]	(*n* = 54) 16.17 (12.85) [*p* = .221]	14.97 (11.00)	(*n* = 69) 17.55 (10.42) [*p* = .013]	(*n* = 60) 14.82 (11.11) [*p* = .807]	14.91 (11.38)	(*n* = 70) 16.14 (12.39) [*p* = .301]	(*n* = 64) 13.90 (10.89) [*p* = .408]	$F(2,259) = 0.90$, $p = .410$, $\eta_p^2 = .007$ [IG = WLC: $p = .316$, $\eta_p^2 = .006$; IG = ACG: $p = .196$, $\eta_p^2 = .009$; ACG = WLC: $p = .202$, $\eta_p^2 = .010$]	$F(2,145) = 1.80$, $p = .169$, $\eta_p^2 = .024$ [IG = WLC: $p = .211$, $\eta_p^2 = .015$; IG = ACG: $p = .523$, $\eta_p^2 = .004$; ACG ≥ WLC: $p = .062$, $\eta_p^2 = .037$]	$F(2,174) = 1.08$, $p = .341$, $\eta_p^2 = .012$ [IG = WLC: $p = .485$, $\eta_p^2 = .004$; IG = ACG: $p = .464$, $\eta_p^2 = .004$; ACG = WLC: $p = .126$, $\eta_p^2 = .020$]
FSS Catastrophizing	15.15 (10.97)	(*n* = 52) 18.81 (11.89) [*p* < .001]	(*n* = 54) 16.17 (12.85) [*p* = .221]	14.97 (11.00)	(*n* = 69) 17.55 (10.42) [*p* = .013]	(*n* = 60) 14.82 (11.11) [*p* = .807]	14.91 (11.38)	(*n* = 70) 16.14 (12.39) [*p* = .301]	(*n* = 64) 13.90 (10.89) [*p* = .408]	$F(2,259) = 0.54$, $p = .586$, $\eta_p^2 = .004$ [IG = WLC: $p = .516$, $\eta_p^2 = .003$; IG = ACG: $p = .769$, $\eta_p^2 = .000$; ACG = WLC: $p = .299$, $\eta_p^2 = .007$]	$F(2,145) = 0.73$, $p = .486$, $\eta_p^2 = .010$ [IG = WLC: $p = .338$, $\eta_p^2 = .009$; IG = ACG: $p = .977$, $\eta_p^2 = .000$; ACG = WLC: $p = .318$, $\eta_p^2 = .011$]	$F(2,174) = 0.57$, $p = .569$, $\eta_p^2 = .006$ [IG = WLC: $p = .511$, $\eta_p^2 = .004$; IG = ACG: $p = .759$, $\eta_p^2 = .001$; ACG = WLC: $p = .308$, $\eta_p^2 = .009$]
FSS Active Coping	15.15 (10.97)	(*n* = 52) 18.81 (11.89) [*p* < .001]	(*n* = 54) 16.17 (12.85) [*p* = .221]	14.97 (11.00)	(*n* = 69) 17.55 (10.42) [*p* = .013]	(*n* = 60) 14.82 (11.11) [*p* = .807]	14.91 (11.38)	(*n* = 70) 16.14 (12.39) [*p* = .301]	(*n* = 64) 13.90 (10.89) [*p* = .408]	$F(2,259) = 0.67$, $p = .674$, $\eta_p^2 = .003$ [IG = WLC: $p = .639$, $\eta_p^2 = .001$;	$F(2,145) = 0.37$, $p = .692$, $\eta_p^2 = .005$ [IG = WLC: $p = .440$, $\eta_p^2 = .006$;	$F(2,174) = 0.16$, $p = .855$, $\eta_p^2 = .002$ [IG = WLC: $p = .669$, $\eta_p^2 = .002$;

	IG = ACG: *p* = .687, η_p^2 = .001; ACG = WLC: *p* = .353, η_p^2 = .005]	IG = ACG: *p* = .645, η_p^2 = .002; ACG = WLC: *p* = .713, η_p^2 = .001]	IG = ACG: *p* = .791, η_p^2 = .001; ACG = WLC: *p* = .700, η_p^2 = .001]

Note. WLC = Waitlist-control group; IG = Intervention group; ACG = Active-control group; Baseline = Baseline assessment; Post = Post Assessment; FU = Follow-up assessment; BDI – II = Beck Depression Inventory; PHQ-9 = Patient Health Questionnaire; PCS Patient Catastrophizing Scale; FABQ = Fear Avoidance Beliefs Questionnaire; FSS = Pain Self-Efficacy Questionnaire; ITT = Intention to treat; PP = Per protocol, CC = Complete cases.

Appendix

Table A. *Complete-case (CC), per-protocol (PP), and intention-to-treat (ITT) analyses across time for primary and secondary outcomes between and within groups from baseline to post intervention*

Baseline to Post Questionnaire Results	WLC			IG			ACG			ITT Baseline to Post (WLC: n =72, IG: n = 97, App: n = 94)	PP Baseline to Post: IG logged in at least once, usage of app (WLC: n = 53, IG: n = 62, App: n = 48)	CC Baseline to Post (WLC: n = 53, IG: n = 69, App: n = 70)
	Baseline	Post (n = 53)	Fu (n = 54)	Baseline	Post (n = 69)	Fu (n = 60)	Baseline	Post (n = 70)	Fu (n = 64)			
BDI-II Total Score	24.96 (9.36)	22.06 (10.59) [p = .008]	18.30 (10.44) [p < .001]	25.35 (10.63)	19.28 (11.64) [p < .001]	16.83 (11.32) [p < .001]	25.00 (9.95)	19.67 (12.05) [p < .001]	20.19 (12.35) [p < .001]	$F(2,259) = 3.23$, p = .041, η_p^2 = .024 [IG > WLC: p = .011, η_p^2 = .039; ACG = IG: p = .485, η_p^2 = .003; App ≥ WLC: p = .078, η_p^2 = .019]	$F(2,159) = 3.63$, p = .029, η_p^2 = .044 [IG > WLC: p = .017, η_p^2 = .049; IG = ACG: p = .792, η_p^2 = .001; ACG > WLC: p = .032, η_p^2 = .046]	$F(2,188) = 2.60$, p =.077, η_p^2 = .027 [IG > WLC: p = .025, η_p^2 = .041; IG = ACG: p = .584, η_p^2 = .002; ACG ≥ WLC: p = .096, η_p^2 = .023]
BDI-II Cognitive Subscale	9.00 (5.02)	(n = 53) 7.72 (5.41) [p = .015]	(n = 54) 6.02 (5.47) [p < .001]	8.65 (5.36)	(n = 69) 6.54 (4.86) [p < .001]	(n = 60) 5.50 (4.88) [p < .001]	8.64 (5.10)	(n = 70) 6.77 (5.56) [p < .001]	(n = 64) 6.95 (5.73) [p = .002]	$F(2,259) = 1.12$, p =.329, η_p^2 = .009 [IG = WLC: p = .130, η_p^2 = .014; ACG = IG: p = .823, η_p^2 = .000; App ≥ WLC: p = .272, η_p^2 = .007]	$F(2,159) = 0.64$, p = .526, η_p^2 = .008 [IG ≥ WLC: p = .088, η_p^2 = .026; IG = ACG: p = .908, η_p^2 = .000; ACG = WLC: p = .164, η_p^2 = .020]	$F(2,188) = 1.10$, p = .334, η_p^2 = .012 [IG = WLC: p = .122, η_p^2 = .020; IG = ACG: p = .672, η_p^2 = .001; ACG = WLC: p = .330, η_p^2 = .008]
BDI-II Somatic-Affective Subscale	15.96 (5.85)	(n = 53) 14.34 (6.46) [p = .021]	(n = 54) 12.28 (6.16) [p < .001]	16.70 (6.19)	(n = 69) 12.74 (7.67) [p < .001]	(n = 60) 11.33 (7.22) [p < .001]	16.33 (6.15)	(n = 70) 12.90 (7.53) [p < .001]	(n = 64) 13.23 (7.66) [p < .001]	$F(2,259) = 3.12$, p =.046, η_p^2 = .024 [IG > WLC: p = .015, η_p^2 = .035; ACG = IG: p = .574, η_p^2 = .002; App ≥ WLC: p = .057, η_p^2 = .022]	$F(2,159) = 3.91$, p =.022, η_p^2 = .047 [IG > WLC: p = .015, η_p^2 = .052; IG = ACG: p = .742, η_p^2 = .001; ACG > WLC: p = .022, η_p^2 = .052]	$F(2,188) = 2.98$, p = .053, η_p^2 = .031 [IG > WLC: p = .020, η_p^2 = .045; IG = ACG: p = .589, η_p^2 = .002; ACG ≥ WLC: p = .061, η_p^2 = .029]
Von Korff Disability Score	56.54 (20.50)	(n = 53) 52.20 (19.90)) [p = .033]	(n = 54) 46.98 (26.69) [p = .001]	54.00 (23.34)	(n = 69) 51.69 (22.89) [p = .119]	(n = 60) 46.67 (22.54) [p = .003]	60.24 (25.89)	(n = 70) 50.43 (23.07) [p < .001]	(n = 64) 52.50 (24.42) [p < .001]	$F(2,259) = 3.05$, p =.049, η_p^2 = .023 [IG = WLC: p = .869, η_p^2 = .000;	$F(2,159) = 2.99$, p = .053, η_p^2 = .036 [IG = WLC: p = .722, η_p^2 = .001;	$F(2,188) = 4.04$, p =.019, η_p^2 = .041 [IG = WLC: p = .509, η_p^2 = .004;

										ACG > IG: p = .022, η_p^2 = .028;<br>ACG ≥ WLC: p = .071, η_p^2 = .020]	ACG > IG: p = .019, η_p^2 = .050;<br>ACG ≥ WLC: p = .079, η_p^2 = .031]	ACG > IG: p = .006, η_p^2 = .054;<br>ACG ≥ WLC: p = .073, η_p^2 = .027]
Von Korff Pain Intensity	2.847 (1.10)	(n = 53)<br>2.74<br>(1.21)<br>[p = .164]	(n = 54)<br>2.52<br>(1.26)<br>[p = .050]	(n = 69)<br>2.88<br>(1.12)	(n = 69)<br>2.83<br>(1.18)<br>[p = .567]	(n = 60)<br>2.52<br>(1.17)<br>[p = .016]	(n = 71)<br>3.06<br>(1.09)	(n = 71)<br>2.70<br>(1.25)<br>[p = .003]	(n = 64)<br>2.90<br>(1.15)<br>[p = .011]	$F(2,259) = 0.21$, p = .207, η_p^2 = .012<br>[IG = WLC: p = .702, η_p^2 = .001;<br>IG = ACG: p = .113, η_p^2 = .013;<br>ACG = WLC: p = .213, η_p^2 = .010]	$F(2,159) = 1.57$, p = .211, η_p^2 = .019<br>[IG = WLC: p = .784, η_p^2 = .001;<br>IG = ACG: p = .130, η_p^2 = .021;<br>ACG = WLC: p = .169, η_p^2 = .019]	$F(2,188) = 0.92$, p =.237, η_p^2 = .015<br>[IG = WLC: p = .579, η_p^2 = .003;<br>IG = ACG: p = .122, η_p^2 = .018;<br>ACG = WLC: p = .289, η_p^2 = .009]
IEQ Total Score	21.30 (10.12)	(n = 53)<br>20.89<br>(9.04)<br>[p = .628]	(n = 54)<br>19.80<br>(10.79)<br>[p = .104]	23.03<br>(9.40)	(n = 69)<br>21.38<br>(10.32)<br>[p = .062]	(n = 60)<br>19.17<br>(9.74)<br>[p < .001]	24.74<br>(10.84)	(n = 70)<br>21.91<br>(10.76)<br>[p < .001]	(n = 64)<br>21.97<br>(10.70)<br>[p < .001]	$F(2,259) = 1.27$, p = .282, η_p^2 = .010<br>[IG = WLC: p = .320, η_p^2 = .006;<br>IG = ACG: p = .510, η_p^2 = .002;<br>ACG ≥ WLC: p = .094, η_p^2 = .017]	$F(2,159) = 0.45$, p = .636, η_p^2 = .006<br>[IG = WLC: p = .446, η_p^2 = .005;<br>IG = ACG: p = .990, η_p^2 = .000;<br>ACG = WLC: p = .380, η_p^2 = .008]	$F(2,188) = 1.12$, p =.329, η_p^2 = .012<br>[IG = WLC: p = .485, η_p^2 = .004;<br>IG = ACG: p = .426, η_p^2 = .005;<br>ACG = WLC: p = .113, η_p^2 = .021]
WHOQOL-BREF (Quality of life global item)	2.71 (0.72)	(n = 52)<br>2.94<br>(0.85)<br>[p = .027]	(n = 54)<br>3.11<br>(0.84)<br>[p = .005]	2.64<br>(0.73)	(n = 69)<br>2.94<br>(0.84)<br>[p = .001]	(n = 60)<br>2.98<br>(0.97)<br>[p = .002]	2.63<br>(0.77)	(n = 70)<br>2.94<br>(0.76)<br>[p < .001]	(n = 64)<br>2.88<br>(0.77)<br>[p = .012]	$F(2,259) = 0.19$, p = .831, η_p^2 = .001<br>[IG = WLC: p = .638, η_p^2 = .001;<br>IG = ACG: p = .940, η_p^2 = .000;<br>ACG = WLC: p = .547, η_p^2 = .002]	$F(2,158) = 0.06$, p = .940, η_p^2 = .001<br>[IG = WLC: p = .768, η_p^2 = .001;<br>IG = ACG: p = .990, η_p^2 = .000;<br>ACG = WLC: p = .760, η_p^2 = .001]	$F(2,187) = 0.12$, p =.889, η_p^2 = .001<br>[IG = WLC: p = .712, η_p^2 = .001;<br>IG = ACG: p = .951, η_p^2 = .000;<br>ACG = WLC: p = .628, η_p^2 = .002]
PHQ-9	12.42 (4.67)	(n = 53)<br>10.78<br>(4.97)<br>[p = .002]	(n = 54)<br>9.96<br>(4.87)<br>[p < .001]	13.52<br>(5.82)	(n = 69)<br>11.06<br>(5.59)<br>[p < .001]	(n = 60)<br>10.05<br>(5.81)<br>[p < .001]	13.36<br>(5.16)	(n = 70)<br>11.44<br>(6.19)<br>[p < .001]	(n = 64)<br>11.49<br>(6.10)<br>[p < .001]	$F(2,259) = 1.33$, p = .265, η_p^2 = .010<br>[IG = WLC: p = .152, η_p^2 = .012;<br>IG = ACG: p = .196, η_p^2 = .009;<br>ACG = WLC: p = .763, η_p^2 = .001]	$F(2,159) = 0.68$, p = .508, η_p^2 = .008<br>[IG = WLC: p = .272, η_p^2 = .011;<br>IG = ACG: p = .445, η_p^2 = .005;<br>ACG = WLC: p = .731, η_p^2 = .001]	$F(2,188) = 0.52$, p =.599, η_p^2 = .005<br>[IG = WLC: p = .404, η_p^2 = .006;<br>IG = ACG: p = .416, η_p^2 = .005;<br>ACG = WLC: p = .833, η_p^2 = .000]

PCS Total Score	22.70 (9.90)	(n = 53)<br>22.66<br>(10.02)<br>[p = .975]	(n = 54)<br>20.59<br>(10.54)<br>[p = .150]	24.39<br>(9.45)	(n = 69)<br>21.68<br>(10.42)<br>[p = .008]	(n = 60)<br>19.80<br>(10.59)<br>[p < .001]	25.10<br>(11.92)	(n = 70)<br>20.61<br>(12.27)<br>[p < .001]	(n = 64)<br>21.47<br>(11.52)<br>[p < .001]	F(2,259) = 3.94, p = .021, η_p^2 = .030<br>[IG ≥ WLC: p = .052, η_p^2 = .022;<br>IG = ACG: p = .384, η_p^2 = .004;<br>ACG > WLC: p = .006, η_p^2 = .045]	F(2,159) = 2.71, p =.069, η_p^2 = .033<br>[IG = WLC: p = .125, η_p^2 = .021;<br>IG = ACG: p = .496, η_p^2 = .004;<br>ACG > WLC: p = .024, η_p^2 = .051]	F(2,188) = 3.77, p =.025, η_p^2 = .039<br>[IG = WLC: p = .139, η_p^2 = .018;<br>IG = ACG: p = .209, η_p^2 = .012;<br>ACG > WLC: p = .006, η_p^2 = .061]
FABQ1 Total Score	9.29 (5.84)	(n = 52)<br>10.58<br>(5.85)<br>[p =.053]	(n = 54)<br>10.30<br>(7.18)<br>[p = .263]	11.31<br>(5.82)	(n = 69)<br>11.28<br>(5.40)<br>[p = .945]	(n = 60)<br>11.65<br>(6.12)<br>[p = .461]	11.19<br>(6.53)	(n = 70)<br>10.31<br>(6.58)<br>[p = .163]	(n = 64)<br>10.61<br>(6.52)<br>[p = .209]	F(2,259) = 2.18, p = .115, η_p^2 = .017<br>[IG = WLC: p = .271, η_p^2 = .007;<br>IG = ACG: p = .289, η_p^2 = .006;<br>ACG > WLC: p = .037, η_p^2 = .026]	F(2,158) = 1.17, p =.313, η_p^2 = .015<br>[IG = WLC: p = .315, η_p^2 = .009;<br>IG = ACG: p = .594, η_p^2 = .003;<br>ACG = WLC: p = .116, η_p^2 = .025]	F(2,187) = 1.61, p =.203, η_p^2 = .017<br>[IG = WLC: p = .533, η_p^2 = .003;<br>IG = ACG: p = .267, η_p^2 = .009;<br>ACG ≥ WLC: p = .068, η_p^2 = .028]
FABQ2 Total Score	15.15<br>(10.97)	(n = 52)<br>18.81<br>(11.89)<br>[p < .001]	(n = 54)<br>16.17<br>(12.85)<br>[p = .221]	14.97<br>(11.00)	(n = 69)<br>17.55<br>(10.42)<br>[p = .013]	(n = 60)<br>14.82<br>(11.11)<br>[p = .807]	14.91<br>(11.38)	(n = 70)<br>16.14<br>(12.39)<br>[p = .301]	(n = 64)<br>13.90<br>(10.89)<br>[p = .408]	F(2,259) = 1.70, p = .186, η_p^2 = .013<br>[IG = WLC: p = .237, η_p^2 = .008;<br>IG = ACG: p = .425, η_p^2 = .003;<br>ACG = WLC: p = .085, η_p^2 = .018]	F(2,158) = 2.55, p =.082, η_p^2 = .031<br>[IG = WLC: p = .134, η_p^2 = .020;<br>IG = ACG: p = .337, η_p^2 = .009;<br>ACG > WLC: p = .037, η_p^2 = .044]	F(2,187) = 1.43, p =.243, η_p^2 = .015<br>[IG = WLC: p = .408, η_p^2 = .006;<br>IG = ACG: p = .345, η_p^2 = .007;<br>ACG = WLC: p = .111, η_p^2 = .021]
FSS<br>Catastrophizing	15.15<br>(10.97)	(n = 52)<br>18.81<br>(11.89)<br>[p < .001]	(n = 54)<br>16.17<br>(12.85)<br>[p = .221]	14.97<br>(11.00)	(n = 69)<br>17.55<br>(10.42)<br>[p = .013]	(n = 60)<br>14.82<br>(11.11)<br>[p = .807]	14.91<br>(11.38)	(n = 70)<br>16.14<br>(12.39)<br>[p = .301]	(n = 64)<br>13.90<br>(10.89)<br>[p = .408]	F(2,259) = 1.10, p = .334, η_p^2 = .008<br>[IG = WLC: p = .161, η_p^2 = .012;<br>IG = ACG: p = .595, η_p^2 = .002;<br>ACG = WLC: p = .312, η_p^2 = .006]	F(2,158) = 1.17, p =.313, η_p^2 = .015<br>[IG = WLC: p = .197, η_p^2 = .015;<br>IG = ACG: p = .714, η_p^2 = .001;<br>ACG = WLC: p = .319, η_p^2 = .010]	F(2,187) = 0.82, p =.441, η_p^2 = .009<br>[IG = WLC: p = .264, η_p^2 = .011;<br>IG = ACG: p = .803, η_p^2 = .000;<br>ACG = WLC: p = .290, η_p^2 = .009]
FSS<br>Active Coping	15.15<br>(10.97)	(n = 52)<br>18.81<br>(11.89)<br>[p < .001]	(n = 54)<br>16.17<br>(12.85)<br>[p = .221]	14.97<br>(11.00)	(n = 69)<br>17.55<br>(10.42)<br>[p = .013]	(n = 60)<br>14.82<br>(11.11)<br>[p = .807]	14.91<br>(11.38)	(n = 70)<br>16.14<br>(12.39)<br>[p = .301]	(n = 64)<br>13.90<br>(10.89)<br>[p = .408]	F(2,259) = 0.16, p = .855, η_p^2 = .001<br>[IG = WLC: p = .624, η_p^2 = .001;	F(2,158) = 0.64, p =.526, η_p^2 = .008<br>[IG = WLC: p = .624, η_p^2 = .002;	F(2,187) = 0.21, p = .811, η_p^2 = .002<br>[IG = WLC: p = .845, η_p^2 = .000;

	IG = ACG: *p* = .917, η_p^2 = .000; ACG = WLC: *p* = .649, η_p^2 = .001]	IG = ACG: *p* = .519, η_p^2 = .004; ACG = WLC: *p* = .253, η_p^2 = .013]	IG = ACG: *p* = .684, η_p^2 = .001; ACG = WLC: *p* = .517, η_p^2 = .004]

Note. WLC = Waitlist-control group; IG = Intervention group; ACG = Active-control group; Baseline = Baseline assessment; Post = Post Assessment; FU = Follow-up assessment; BDI – II = Beck Depression Inventory; PHQ-9 = Patient Health Questionnaire; PCS Patient Catastrophizing Scale; FABQ = Fear Avoidance Beliefs Questionnaire; FSS = Pain Self-Efficacy Questionnaire; ITT = Intention to treat; PP = Per protocol, CC = Complete cases.

Table B. *Complete-case (CC), per-protocol (PP), and intention-to-treat (ITT) analyses across time for primary and secondary outcomes between and within groups from baseline to follow-up intervention*

Baseline to Follow-Up Questionnaire Results	WLC			IG			ACG			ITT Baseline to FU (WLC: $n = 72$, IG: $n = 97$, ACG: $n = 94$)	PP Baseline to FU: IG logged in at least once, usage of ACG (WLC: $n = 51$, IG: $n = 54$, ACG: $n = 44$)	CC: Baseline to FU (WLC: $n = 54$, IG: $n = 60$, ACG: $n = 64$)
	Baseline	Post ($n = 53$)	FU ($n = 54$)	Baseline	Post ($n = 69$)	FU ($n = 60$)	Baseline	Post ($n = 70$)	FU ($n = 64$)			
BDI-II Total Score	24.96 (9.36)	22.06 (10.59) [$p = .008$]	18.30 (10.44) [$p < .001$]	25.35 (10.63)	19.28 (11.64) [$p < .001$]	16.83 (11.32) [$p < .001$]	25.00 (9.95)	19.67 (12.05) [$p < .001$]	20.19 (12.35) [$p < .001$]	$F(2,259) = 3.75$, $p = .025$, $\eta_p^2 = .028$<br>[IG ≥ WLC: $p = .072$, $\eta_p^2 = .019$; IG > ACG: $p = .011$, $\eta_p^2 = .034$; ACG = WLC: $p = .452$, $\eta_p^2 = .003$]	$F(2,145) = 1.41$, $p = .248$, $\eta_p^2 = .019$<br>[IG = WLC: $p = .199$, $\eta_p^2 = .016$; IG = ACG: $p = .146$, $\eta_p^2 = .022$; ACG = WLC: $p = .803$, $\eta_p^2 = .001$]	$F(2,174) = 3.90$, $p = .022$, $\eta_p^2 = .043$<br>[IG = WLC: $p = .123$, $\eta_p^2 = .021$; IG > ACG: $p = .010$, $\eta_p^2 = .054$; ACG = WLC: $p = .234$, $\eta_p^2 = .012$]
BDI-II Cognitive Subscale	9.00 (5.02)	($n = 53$) 7.72 (5.41) [$p = .015$]	($n = 54$) 6.02 (5.47) [$p < .001$]	8.65 (5.36)	($n = 69$) 6.54 (4.86) [$p < .001$]	($n = 60$) 5.50 (4.88) [$p < .001$]	8.64 (5.10)	($n = 70$) 6.77 (5.56) [$p < .001$]	($n = 64$) 6.95 (5.73) [$p = .002$]	$F(2,259) = 1.96$, $p = .143$, $\eta_p^2 = .015$<br>[IG = WLC: $p = .389$, $\eta_p^2 = .004$; IG ≥ ACG: $p = .051$, $\eta_p^2 = .020$; ACG = WLC: $p = .314$, $\eta_p^2 = .006$]	$F(2,145) = 0.81$, $p =.447$, $\eta_p^2 = .011$<br>[IG = WLC: $p = .506$, $\eta_p^2 = .004$; IG = ACG: $p = .220$, $\eta_p^2 = .016$; ACG = WLC: $p = .517$, $\eta_p^2 = .005$]	$F(2,174) = 2.95$, $p = .055$, $\eta_p^2 = .033$<br>[IG = WLC: $p = .326$, $\eta_p^2 = .009$; IG > ACG: $p = .021$, $\eta_p^2 = .043$; ACG = WLC: $p = .173$, $\eta_p^2 = .016$]
BDI-II Somatic-Affective Subscale	15.96 (5.85)	($n = 53$) 14.34 (6.46) [$p = .021$]	($n = 54$) 12.28 (6.16) [$p < .001$]	16.70 (6.19)	($n = 69$) 12.74 (7.67) [$p < .001$]	($n = 60$) 11.33 (7.22) [$p < .001$]	16.33 (6.15)	($n = 70$) 12.90 (7.53) [$p < .001$]	($n = 64$) 13.23 (7.66) [$p < .001$]	$F(2,259) = 3.45$, $p = .032$, $\eta_p^2 = .026$<br>[IG > WLC: $p = .050$, $\eta_p^2 = .023$; IG > ACG: $p = .018$, $\eta_p^2 = .030$; ACG ≥ WLC: $p = .057$, $\eta_p^2 = .022$]	$F(2,145) = 1.64$, $p =.197$, $\eta_p^2 = .022$<br>[IG = WLC: $p = .112$, $\eta_p^2 = .025$; IG = ACG: $p = .164$, $\eta_p^2 = .020$; ACG = WLC: $p = .913$, $\eta_p^2 = .000$]	$F(2,174) = 3.64$, $p = .028$, $\eta_p^2 = .040$<br>[IG ≥ WLC: $p = .083$, $\eta_p^2 = .027$; IG > ACG: $p = .014$, $\eta_p^2 = .049$; ACG = WLC: $p = .379$, $\eta_p^2 = .007$]
Von Korff Disability Score	56.54 (20.50)	($n = 53$) 52.20 (19.90)) [$p = .033$]	($n = 54$) 46.98 (26.69) [$p = .001$]	54.00 (23.34)	($n = 69$) 51.69 (22.89) [$p = .119$]	($n = 60$) 46.67 (22.54) [$p = .003$]	60.24 (25.89)	($n = 70$) 50.43 (23.07) [$p < .001$]	($n = 64$) 52.50 (24.42) [$p < .001$]	$F(2,259) = 0.03$, $p =.968$, $\eta_p^2 = .000$<br>[IG = WLC: $p = .807$, $\eta_p^2 = .000$;	$F(2,145) = 0.34$, $p = .711$, $\eta_p^2 = .005$<br>[IG = WLC: $p = .760$, $\eta_p^2 = .001$;	$F(2,174) = 0.23$, $p = .794$, $\eta_p^2 = .003$<br>[IG = WLC: $p = .557$, $\eta_p^2 = .003$;

										IG = ACG: p = .973, η_p^2 = .000;<br>ACG = WLC: p = .836, η_p^2 = .000]	IG = ACG: p = .538, η_p^2 = .004;<br>ACG = WLC: p = .450, η_p^2 = .006]	IG = ACG: p = .884, η_p^2 = .000;<br>ACG = WLC: p = .587, η_p^2 = .003]
Von Korff Pain Intensity	2.847 (1.10)	(n = 53)<br>2.74 (1.21)<br>[p = .164]	(n = 54)<br>2.52 (1.26)<br>[p = .050]	(n = 69)<br>2.88 (1.12)	(n = 69)<br>2.83 (1.18)<br>[p = .567]	(n = 60)<br>2.52 (1.17)<br>[p = .016]	(n = 71)<br>3.06 (1.09)	(n = 71)<br>2.70 (1.25)<br>[p = .003]	(n = 64)<br>2.90 (1.15)<br>[p = .011]	$F(2,259) = 0.52$, p =.598, η_p^2 = .004<br>[IG = WLC: p = .648, η_p^2 = .001;<br>IG = ACG: p = .290, η_p^2 = .006;<br>ACG = WLC: p = .655, η_p^2 = .001]	$F(2,145) = 0.23$, p = .798, η_p^2 = .003<br>[IG = WLC: p = .792, η_p^2 = .001;<br>IG = ACG: p = .573, η_p^2 = .003;<br>ACG = WLC: p = .707, η_p^2 = .002]	$F(2,174) = 0.32$, p = .730, η_p^2 = .004<br>[IG = WLC: p = .835, η_p^2 = .000;<br>IG = ACG: p = .420, η_p^2 = .005;<br>ACG = WLC: p = .661, η_p^2 = .002]
IEQ Total Score	21.30 (10.12)	(n = 53)<br>20.89 (9.04)<br>[p = .628]	(n = 54)<br>19.80 (10.79)<br>[p = .104]	23.03 (9.40)	(n = 69)<br>21.38 (10.32)<br>[p = .062]	(n = 60)<br>19.17 (9.74)<br>[p < .001]	24.74 (10.84)	(n = 70)<br>21.91 (10.76)<br>[p < .001]	(n = 64)<br>21.97 (10.70)<br>[p < .001]	$F(2,259) = 1.18$, p = .309, η_p^2 = .009<br>[IG = WLC: p = .128, η_p^2 = .014;<br>IG = ACG: p = .401, η_p^2 = .004;<br>ACG = WLC: p = .479, η_p^2 = .003]	$F(2,145) = 1.63$, p = .199, η_p^2 = .022<br>[IG = WLC: p = .112, η_p^2 = .025;<br>IG = ACG: p = .149, η_p^2 = .022;<br>ACG = WLC: p = .831, η_p^2 = .001]	$F(2,174) = 1.92$, p = .149, η_p^2 = .022<br>[IG ≥ WLC: p = .060, η_p^2 = .032;<br>IG = ACG: p = .197, η_p^2 = .014;<br>ACG = WLC: p = .486, η_p^2 = .004]
WHOQOL-BREF (Quality of life global item)	2.71 (0.72)	(n = 52)<br>2.94 (0.85)<br>[p = .027]	(n = 54)<br>3.11 (0.84)<br>[p = .005]	2.64 (0.73)	(n = 69)<br>2.94 (0.84)<br>[p = .001]	(n = 60)<br>2.98 (0.97)<br>[p = .002]	2.63 (0.77)	(n = 70)<br>2.94 (0.76)<br>[p < .001]	(n = 64)<br>2.88 (0.77)<br>[p = .012]	$F(2,259) = 0.86$, p = .426, η_p^2 = .007<br>[IG = WLC: p = .855, η_p^2 = .000;<br>IG = ACG: p = .282, η_p^2 = .006;<br>ACG = WLC: p = .208, η_p^2 = .010]	$F(2,145) = 0.72$, p = .487, η_p^2 = .010<br>[IG = WLC: p = .898, η_p^2 = .000;<br>IG = ACG: p = .318, η_p^2 = .011;<br>ACG = WLC: p = .234, η_p^2 = .015]	$F(2,174) = 0.84$, p = .435, η_p^2 = .010<br>[IG = WLC: p = .782, η_p^2 = .001;<br>IG = ACG: p = .335, η_p^2 = .008;<br>ACG = WLC: p = .183, η_p^2 = .015]
PHQ-9	12.42 (4.67)	(n = 53)<br>10.78 (4.97)<br>[p = .002]	(n = 54)<br>9.96 (4.87)<br>[p < .001]	13.52 (5.82)	(n = 69)<br>11.06 (5.59)<br>[p < .001]	(n = 60)<br>10.05 (5.81)<br>[p < .001]	13.36 (5.16)	(n = 70)<br>11.44 (6.19)<br>[p < .001]	(n = 64)<br>11.49 (6.10)<br>[p < .001]	$F(2,259) = 2.91$, p = .056, η_p^2 = .022<br>[IG = WLC: p = .167, η_p^2 = .011;<br>IG > ACG: p = .022, η_p^2 = .028;<br>ACG = WLC: p = .530, η_p^2 = .002]	$F(2,145) = 1.80$, p = .169, η_p^2 = .024<br>[IG = WLC: p = .230, η_p^2 = .014;<br>IG ≥ ACG: p = .087, η_p^2 = .031;<br>ACG = WLC: p = .656, η_p^2 = .002]	$F(2,174) = 2.64$, p = .075, η_p^2 = .029<br>[IG = WLC: p = .219, η_p^2 = .014;<br>IG > ACG: p = .032, η_p^2 = .037;<br>ACG = WLC: p = .487, η_p^2 = .004]

PCS Total Score	22.70 (9.90)	(*n* = 53) 22.66 (10.02) [*p* = .975]	(*n* = 54) 20.59 (10.54) [*p* = .150]	24.39 (9.45)	(*n* = 69) 21.68 (10.42) [*p* = .008]	(*n* = 60) 19.80 (10.59) [*p* < .001]	25.10 (11.92)	(*n* = 70) 20.61 (12.27) [*p* < .001]	(*n* = 64) 21.47 (11.52) [*p* < .001]	*F*(2,259) = 2.0, *p* = .139, η_p^2 = .015<br>[IG ≥ WLC: *p* = .052, η_p^2 = .023; IG = ACG: *p* = .366, η_p^2 = .004; ACG = WLC: *p* = .243, η_p^2 = .008]	*F*(2,145) = 1.41, *p* = .300, η_p^2 = .016<br>[IG = WLC: *p* = .152, η_p^2 = .020; IG = ACG: *p* = .426, η_p^2 = .007; ACG = WLC: *p* = .459, η_p^2 = .006]	*F*(2,174) = 1.43, *p* = .243, η_p^2 = .016<br>[IG = WLC: *p* = .116, η_p^2 = .022; IG = ACG: *p* = .506, η_p^2 = .004; ACG = WLC: *p* = .268, η_p^2 = .011]
FABQ1 Total Score	9.29 (5.84)	(*n* = 52) 10.58 (5.85) [*p* =.053]	(*n* = 54) 10.30 (7.18) [*p* = .263]	11.31 (5.82)	(*n* = 69) 11.28 (5.40) [*p* = .945]	(*n* = 60) 11.65 (6.12) [*p* = .461]	11.19 (6.53)	(*n* = 70) 10.31 (6.58) [*p* = .163]	(*n* = 64) 10.61 (6.52) [*p* = .209]	*F*(2,259) = 1.10, *p* = .338, η_p^2 = .008<br>[IG = WLC: *p* = .753, η_p^2 = .001; IG = ACG: *p* = .244, η_p^2 = .007; ACG = WLC: *p* = .166, η_p^2 = .012]	*F*(2,145) = 1.22, *p* = .299, η_p^2 = .017<br>[IG = WLC: *p* = .883, η_p^2 = .000; IG = ACG: *p* = .177, η_p^2 = .019; ACG = WLC: *p* = .151, η_p^2 = .022]	*F*(2,174) = 1.16, *p* = .315, η_p^2 = .013<br>[IG = WLC: *p* = .928, η_p^2 = .000; IG = ACG: *p* = .156, η_p^2 = .017; ACG = WLC: *p* = .205, η_p^2 = .014]
FABQ2 Total Score	15.15 (10.97)	(*n* = 52) 18.81 (11.89) [*p* < .001]	(*n* = 54) 16.17 (12.85) [*p* = .221]	14.97 (11.00)	(*n* = 69) 17.55 (10.42) [*p* = .013]	(*n* = 60) 14.82 (11.11) [*p* = .807]	14.91 (11.38)	(*n* = 70) 16.14 (12.39) [*p* = .301]	(*n* = 64) 13.90 (10.89) [*p* = .408]	*F*(2,259) = 0.90, *p* = .410, η_p^2 = .007<br>[IG = WLC: *p* = .316, η_p^2 = .006; IG = ACG: *p* = .196, η_p^2 = .009; ACG = WLC: *p* = .202, η_p^2 = .010]	*F*(2,145) = 1.80, *p* = .169, η_p^2 = .024<br>[IG = WLC: *p* = .211, η_p^2 = .015; IG = ACG: *p* = .523, η_p^2 = .004; ACG ≥ WLC: *p* = .062, η_p^2 = .037]	*F*(2,174) = 1.08, *p* = .341, η_p^2 = .012<br>[IG = WLC: *p* = .485, η_p^2 = .004; IG = ACG: *p* = .464, η_p^2 = .004; ACG = WLC: *p* = .126, η_p^2 = .020]
FSS Catastrophizing	15.15 (10.97)	(*n* = 52) 18.81 (11.89) [*p* < .001]	(*n* = 54) 16.17 (12.85) [*p* = .221]	14.97 (11.00)	(*n* = 69) 17.55 (10.42) [*p* = .013]	(*n* = 60) 14.82 (11.11) [*p* = .807]	14.91 (11.38)	(*n* = 70) 16.14 (12.39) [*p* = .301]	(*n* = 64) 13.90 (10.89) [*p* = .408]	*F*(2,259) = 0.54, *p* = .586, η_p^2 = .004<br>[IG = WLC: *p* = .516, η_p^2 = .003; IG = ACG: *p* = .769, η_p^2 = .000; ACG = WLC: *p* = .299, η_p^2 = .007]	*F*(2,145) = 0.73, *p* = .486, η_p^2 = .010<br>[IG = WLC: *p* = .338, η_p^2 = .009; IG = ACG: *p* = .977, η_p^2 = .000; ACG = WLC: *p* = .318, η_p^2 = .011]	*F*(2,174) = 0.57, *p* = .569, η_p^2 = .006<br>[IG = WLC: *p* = .511, η_p^2 = .004; IG = ACG: *p* = .759, η_p^2 = .001; ACG = WLC: *p* = .308, η_p^2 = .009]
FSS Active Coping	15.15 (10.97)	(*n* = 52) 18.81 (11.89) [*p* < .001]	(*n* = 54) 16.17 (12.85) [*p* = .221]	14.97 (11.00)	(*n* = 69) 17.55 (10.42) [*p* = .013]	(*n* = 60) 14.82 (11.11) [*p* = .807]	14.91 (11.38)	(*n* = 70) 16.14 (12.39) [*p* = .301]	(*n* = 64) 13.90 (10.89) [*p* = .408]	*F*(2,259) = 0.67, *p* = .674, η_p^2 = .003<br>[IG = WLC: *p* = .639, η_p^2 = .001;	*F*(2,145) = 0.37, *p* = .692, η_p^2 = .005<br>[IG = WLC: *p* = .440, η_p^2 = .006;	*F*(2,174) = 0.16, *p* = .855, η_p^2 = .002<br>[IG = WLC: *p* = .669, η_p^2 = .002;

	IG = ACG: p = .687, η_p^2 = .001; ACG = WLC: p = .353, η_p^2 = .005]	IG = ACG: p = .645, η_p^2 = .002; ACG = WLC: p = .713, η_p^2 = .001]	IG = ACG: p = .791, η_p^2 = .001; ACG = WLC: p = .700, η_p^2 = .001]

Note. WLC = Waitlist-control group; IG = Intervention group; ACG = Active-control group; Baseline = Baseline assessment; Post = Post Assessment; FU = Follow-up assessment; BDI – II = Beck Depression Inventory; PHQ-9 = Patient Health Questionnaire; PCS Patient Catastrophizing Scale; FABQ = Fear Avoidance Beliefs Questionnaire; FSS = Pain Self-Efficacy Questionnaire; ITT = Intention to treat; PP = Per protocol, CC = Complete cases.
